# London Education and Inclusion Project (LEIP): Exploring Negative and Null Effects of a Cluster-Randomised School-Intervention to Reduce School Exclusion—Findings from Protocol-Based Subgroup Analyses

**DOI:** 10.1371/journal.pone.0152423

**Published:** 2016-04-05

**Authors:** Ingrid Obsuth, Aiden Cope, Alex Sutherland, Liv Pilbeam, Aja Louise Murray, Manuel Eisner

**Affiliations:** Institute of Criminology, University of Cambridge, Cambridge, United Kingdom; TNO, NETHERLANDS

## Abstract

This paper presents subgroup analyses from the London Education and Inclusion Project (LEIP). LEIP was a cluster-randomised controlled trial of an intervention called Engage in Education-London (EiE-L) which aimed to reduce school exclusions in those at greatest risk of exclusion. Pupils in the control schools attended an hour-long employability seminar. Minimisation was used to randomly assign schools to treatment and control following baseline data collection. The study involved 36 schools (17 in treatment—373 pupils; 19 in control—369 pupils) with >28% free school meal eligibility across London and utilised on pupil self-reports, teacher reports as well as official records to assess the effectiveness of EiE-L. Due to multiple data sources, sample sizes varied according to analysis. Analyses of pre-specified subgroups revealed null and negative effects on school exclusion following the intervention. Our findings suggest that the design and implementation of EiE-L may have contributed to the negative outcomes for pupils in the treatment schools when compared to those in the control schools. These findings call into question the effectiveness of bolt-on short-term interventions with pupils, particularly those at the highest risk of school exclusion and when they are faced with multiple problems. This is especially pertinent given the possibility of negative outcomes.

***Trial Registration***: Controlled Trials: ISRCTN23244695

## Introduction

School based interventions that target problem behaviours are common but are rarely evaluated in a comprehensive manner. Existing evaluations have focused on overall treatment effects (or main effects). However, in order to better understand the effects of an intervention, it is necessary to go beyond whether it works overall and examine what works, for whom and under what conditions (e.g. [1–3]). Such analyses are also useful and particularly pertinent when negative or null main effects are identified, as they may provide insights into these effects. Moreover, given the potential for harm, eliminating interventions with negative effects should be a priority for all. In this paper we explore the possibility that the Engage in Education—London (EiE-L) programme resulted in differential effects on school exclusions based on a set of pre-specified characteristics.

EiE-L was evaluated as part of the London Education and Inclusion Project (LEIP). LEIP is a cluster-randomised controlled trial involving 36 London secondary schools. EiE-L aimed to reduce fixed-term school exclusions by targeting the interpersonal communication and broader social skills of young people identified by teachers as being most at risk of being excluded (see published study protocol, [4]). Intent-to-treat analyses of treatment main effects revealed that young people in treatment schools self-reported a *higher* occurrence of school exclusions than those in the control schools. No further significant differences were observed between the young people in the two groups on any secondary outcomes relating to interpersonal, behavioural or educational domains [5].

### Background to the project

The trial was designed and implemented by a research team at the Institute of Criminology, University of Cambridge in collaboration with the Greater London Authority and was funded by the European Commission. The intervention was funded by the Education Endowment Foundation (EEF). Of the providers responding to a call by the EEF for interventions that could reduce school exclusion, the developers of EiE-L offered the clearest description of aims, and had promising findings from a preliminary evaluation [6]. EiE-L was a 12-week-long programme developed by two UK non-profit organisations. The intervention consisted of weekly group and one-to-one sessions delivered to young people in Years 9 and 10. Young people in control schools received a one-off hour-long employability skills workshop [4].

The intervention targeted interpersonal communication and broader social skills as the mechanisms of change, with the expectation that improvements in these skills would reduce disruptive or antisocial behaviour and thus exclusions. Research suggests that there is a link between communication and broader social skills difficulties and behaviours leading to exclusion [7, 8]. The majority of exclusions in the UK (around 50%) are in response to verbal abuse or physical assault by pupils. However, the most commonly cited single reason for exclusion is “persistent disruptive behaviour” [9] suggesting that young people are often excluded for relatively minor infractions. Using exclusion to deal with behavioural problems may be counter productive as it is associated with a range of negative outcomes, including problem behaviour [10–13]. EiE-L was evaluated in the hope that it could offer a feasbile way of diverting young people from being excluded.

### The current study

In this paper we examine whether EiE-L resulted in differential effects on young people based on pre-specified treatment and individual characteristics [4]. Our analyses focused on the potential moderating effects that these characteristics may have on the link between EiE-L and our primary outcome of fixed-term school exclusion. We report results based on pupil self-reports, teacher-reports as well as official records of exclusion. The knowledge that the intervention overall led to increased self-reported exclusions highlights the importance of carrying out further analyses. These were utilised to gain insight into the reasons underlying these overall iatrogenic effects.

We selected (i.e. pre-specified) our sub-groups based on previous research suggesting that several factors may influence treatment effects in a school context. The three broad areas of the sub-groups were: treatment characteristics (attendance and engagement), individual baseline characteristics (behaviour, communication, school bond, student-teacher relationship), and demographic characteristics (sex and school year).

#### Treatment characteristics: programme attendance (“dose”) and engagement

Previous research has identified intervention attendance (or dosage) and treatment engagement as key characteristics when examining implementation quality. These treatment characteristics have also been identified as important predictors of differential programme effectiveness (e.g. [2, 14, 15–18]). Implementation quality has been argued to play a pivotal role in the success of interventions [19] and programmes with high implementation quality have been shown to yield greater effects sizes than programmes with implementation problems [20]. We hypothesised that pupils with higher attendance and greater engagement would have better outcomes following the intervention than pupils with low attendance or poorer engagement when compared to controls.

#### Individual baseline characteristics

Previous research suggests that interventions are most effective when baseline problems are high enough to enable the possibility of a meaningful change (e.g. [2, 21]). However, others (e.g. [22]) suggest that having *fewer* baseline problems may enable participants to gain greater benefits from interventions. To explore these possibilities, we carried out subgroup analyses for baseline levels of both anti-social behaviour and communication skills.

Extensive literature also shows that adolescents’ bond or connectedness to school and positive relationships with teachers have beneficial effects on their overall development and behaviours [23–26]. Excluding young people who display difficult behaviour at school may be counterproductive, as an already weak school bond may be weakened further, limiting the potential protective effect of schooling [27]. Research by Pomeroy [28] also highlights that excludees identify a positive student-teacher relationship as one of the key aspects in their motivation to remain in education. For these reasons we expected that pupil’s engagement with the school and their teachers would also influence how they responded to the intervention.

#### Demographic characteristics

National statistics from the UK show that pupil attributes are strongly associated with the likelihood of exclusion [9]. Boys are two to three times more likely to receive school exclusion than girls between the ages of 11–16. The prevalence of exclusion also peaks for both sexes between the ages of 13–15, corresponding to Year 9 and Year 10 in the UK. Given the much higher prevalence of exclusion for boys and for older pupils, we examined whether the effects of the intervention differed for boys and girls and in the different year groups in the treatment versus control groups.

## Materials and Methods

### Ethics statement

This study and its procedures were approved by the Ethics Committee of the Institute of Criminology, University of Cambridge on 20 May 2013, and follows the rules stated in the Declaration of Helsinki. All schools involved in the study signed data sharing agreements with the University of Cambridge. After identification of the young people, passive informed consent (“opt out”) was sought and obtained from parents via the following procedure: Letters were drafted by the research team and were amended and sent by participating schools. Parents were given one week to advise the research team by contacting the school (either by post or phone) and indicating that they wish to opt their child out of the study; 26 parents/guardians opted their child out of the study and were not approached to participate by the research team. Assent was also sought from the participating young people. Thirteen young people did not assent to data collection, their information was not used in any subsequent analyses.

### Project overview

LEIP was a cRCT that took place in 36 secondary schools in London. To qualify for the trial, schools had to have 28% or more of their pupils eligible for free school meals (a means tested benefit). Schools were recuirted from May to September 2013 by the research and intervention team members. Participating schools were asked to identify 10–12 pupils in Years 9 and 10 (20–24 in total) who were at greatest risk for fixed-term school exclusion according to criteria provided by the evaluation team—i.e. based on persistent absences, previous exclusion or behaviours likely to lead to exclusion. Baseline data collection (teacher and young person questionnaires) was completed from June—October 2013 (teacher reports) and from September—October 2013 (young person reports). Randomisation took place in Autumn 2013 following completion of baseline data collection.

Schools were randomised by a member of the research team via minimisation. Minimisation [29] was used to assign schools to the treatment versus control condition. Allocation was undertaken using the MinimPy software developed by Saghaei and Saghaei [30] based on balancing factors previously identified in other research as being associated with an increased likelihood of exclusion: free school meal eligibility, special educational need (SEN) status, school size and school composition (mixed vs. single sex), and teacher reported baseline behaviour problems [4]. This process was selected as it offers several advantages over pure random allocation, some have even argued it is the “platinum standard” for randomisation [31]. The essence of the minimisation approach is that it does not rely solely on chance—it aims to reduce (i.e. minimise) differences in determinants of the outcome so that any remaining differences can be attributed to the outcome. To overcome the issue that pure minimisation is deterministic, the algorithms used also include a random component that reduces the chance of prediction—rather than favouring a reduction in imbalance scores, preference is given to allocation to treatment [31]. Thus, the minimisation algorithm is a flexible allocation method in which the allocation of each subject (e.g. individual or school) is influenced by the existing overall balance of allocated subjects [4]. One consequence of focusing on balance is that minimisation can lead to unequal sample sizes in treatment allocation arms. Nineteen schools were randomised into the control condition and 17 into the treatment condition. The treatment schools received the EiE-L intervention in Autumn 2013 (10 schools, Phase I) or in Winter/Spring 2014 (7 schools, Phase II).

**Fig 1:**
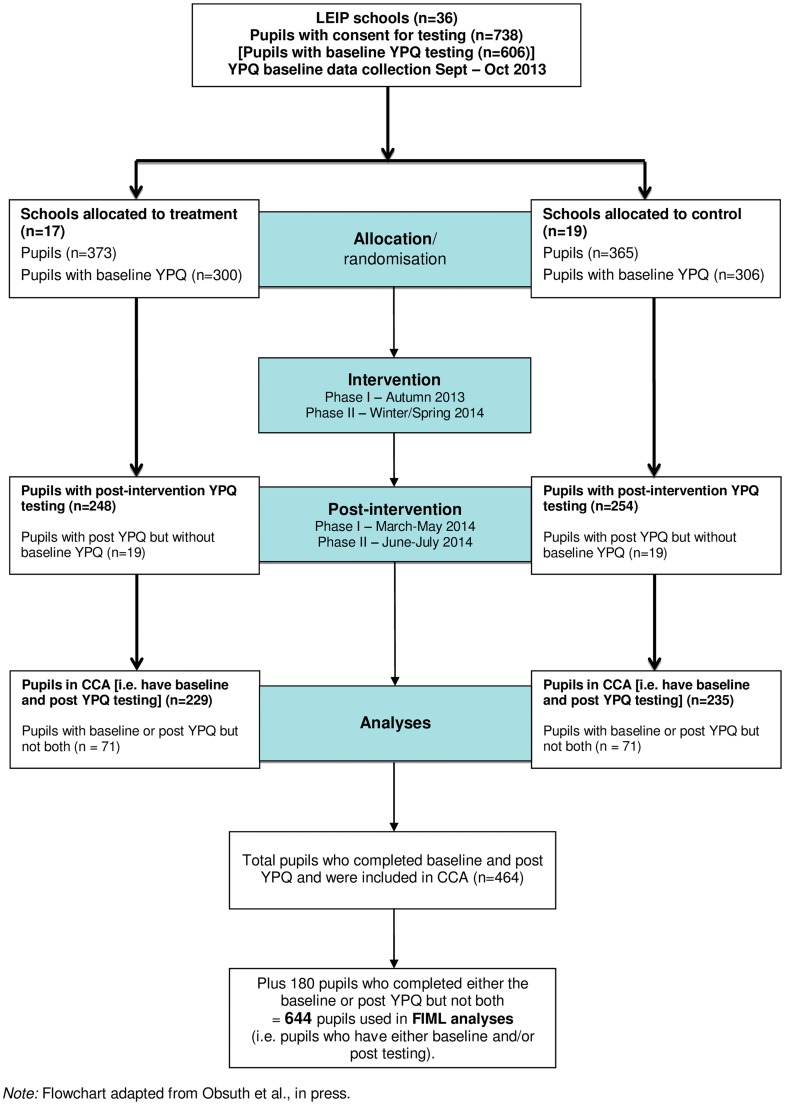
CONSORT Flowchart.

Post-intervention data collection was completed one month after completion of the intervention in each school; from March—May 2014 in Phase I schools and June—July 2014 in Phase II schools. See Fig 1 for CONSORT flowchart. The study was registered after enrolment of participants started as at the intiaiton of recuriment it had not yet become common practice to register trials evaluating psycho-social interventions. Registration of trails in social sciences is an essential but relatively new development. The protocol for this trial and CONSORT checklist are available as supporting information; see S1 CONSORT Checklist and S1 Protocol.

### EiE-L intervention

As above, the intervention consisted of group and individual sessions with pupils. The intervention with pupils was delivered by two “core workers” per school, who were trained and employed specifically for this task by the intervention provider. Group sessions, attended by up to twelve pupils, were structured around specific goals such as learning to co-operate with others, managing difficult emotions or communication through body language and facial expression. Pupils either worked in pairs or participated in whole group discussions during group sessions. One-to-one sessions with pupils built on group work by adapting the goals to each young person’s needs. The intervention as planned also included home-visits and telephone calls to participants and their family. This resulted in eleven home-visits and 164 telephone calls being made. In addition, one of the partner organisations, which specialises in communication difficulties in children, provided material for group sessions and delivered training to selected school staff on how to identify and support young people with communication needs. This took the form of two workshops and follow-up sessions with teachers. The authors confirm that all ongoing and related trials for this intervention are registered.

### The control group

Schools in the control group were offered a one-off one-hour-long workshop delivered by trained corporate volunteers. These workshop sessions addressed employability skills of young people, provided insight into the world of work and facilitated discussions concerning employment.

### Power analysis

Due to funding restraints our maximum sample was fixed to 40 schools and up to 24 young people per school (see protocol for further details, [4]). We conducted a Monte Carlo power analysis to evaluate the power to detect moderation effects given our target and anticipated sample size (see Power Analysis in S1 Text and Table A in S1 Text). The results suggested that there was good power to detect a moderation effect equivalent to a standardised regression coefficient of >.20 but a lack of significant moderation would not be strong evidence against the presence of moderation effects smaller than this.

### Sample

The study sample consisted of young people for whom data was available either from baseline and/or post-intervention data collection (*n* = 644 for young person reports; *n* = 689 for teacher reports). Details about the treatment of missing data are provided below. Seventy-one percent of the sample were male. Forty percent of pupils in the sample self-identified as “Black-African, Black-Caribbean or Black British” (*n* = 244), 25% identified as “White British” (*n* = 151; see [5]).

#### Analyses sample sizes

To make use of all available information, separate analyses were carried out for young person and teacher reported data. This means that sample sizes differ for each source. The complete case analysis (CCA) samples consisted of young people with complete data at baseline *and* post-intervention assessment. Analyses utilising Full Information Maximum Likelihood (FIML) estimation (further discussed below) consisted of young people for whom data was available from *either* baseline *or* post-intervention data collection. Official records were analysed based on the information requested from the Department for Education, United Kingdom (DfE). These analyses were completed based on the sample for whom we requested data and were carried out on CCA basis only. Table 1 outlines the sample size for each data source and analysis. Fig 1 and Table 2 outline participant flow and reasons for non-completion of post-intervention YPQs, i.e. missing data.

**Table 1:** Maximum Analysis Sample Sizes by Analysis Type and Data Source.

	Analysis sample size: pupils (schools)	
Data source	Complete cases analysis	FIML analysis
Young person exclusion	*n* = 464 (35)	*n* = 644 (36)
Teacher exclusion	*n* = 424 (34)	*n* = 685 (36)
Official record of exclusion	*n* = 710 (36)	n/a

**Table 2:** Reasons for Non-completion of Post-intervention YPQ for Young People with Baseline YPQ (i.e. from *n* = 606 young people).

	Treatment	Control
Opted out by parents	2	1
Child refused to participate	3	5
Opted out by school	5	14
Child was asked to leave school via permanent exclusion or managed move	14	12
Left school	30	25
Child not available on 3+ attempts	17	14
**Total**	71	71

### Measures

Details about the construction and reliability of the study measures are given in Obsuth et al. [5], so we offer brief descriptions here. We assessed inter-item reliability and report Cronbach’s alpha (*α*) for each measure. Young people and teachers completed the same questionnaire at baseline testing and following the intervention. The reference period for baseline and post-intervention testing differed due to time constraints. Baseline testing asked young people and teachers to report based on the previous nine months (“since January”), whereas post-intervention testing was reported based on the “previous four weeks” (the four weeks following completion of the intervention). The intervention provider also provided documents for each group and one-to-one session—referred to as a session plan summary (SPS). The SPS summarised the planned content of sessions, provided rating scales to assess behaviours in sessions, time spent on task, and relevant notes. These were utilised to assess implementation quality.

#### Primary Outcome

*School exclusion*—Young people and teachers completed questions asking about the frequency of 14 different school disciplinary measures each rated on a six-point scale ranging from “never” to “every day”. Two questions covered the frequency of “fixed-term exclusion” and “suspensions”. We included both terms as they are commonly used in practice, but not always interchangeably. These were used to create a dichotomous outcome of “excluded” or “not excluded”, where *any* exclusion *or* suspension was coded “1” and those reporting “never” to both questions were coded as “not excluded”.

Official records of school exclusions from the National Pupil Database (NPD) of the DfE, UK were also requested. The NPD is a census of all school pupils in England. Data on exclusions are collected by schools for every child for each term of the school year, with schools asked to specify the type of exclusion and (if applicable) the length of exclusion and the date(s) the exclusion took place. This information is then passed on to the DfE who release aggregated data on exclusions annually [10]. We requested NPD data for 714 pupils who had consented to allow official records to be requested. Data were returned for 260 pupils who were reported by their school to have experienced at least one fixed term exclusion in the 2013/2014 academic year. Of those only 57 (35 in treatment group; 22 in control group) young people experienced at least one fixed term exclusion during the post-intervention period. This period was set to be six weeks following the completion of the intervention in each Phase; it constituted the maximum amount of time between the end of the intervention in Phase II schools in May 2014 and the end of the school year.

#### Measures for subgroups

**Treatment characteristics**: *Attendance (“dose”)*: Treatment dosage, operationalised as attendance, was recorded for all one-to-one and group sessions. Descriptive statistics for attendance were based on the sample of young people in the treatment schools for whom we had baseline and/or post-intervention data (*n* = 320). Of those 320 young people, 273 attended at least one (of 12) group sessions (*M* = 6.85; *sd* = 3.96); 280 attended at least one of 12 one-to-one sessions (*M* = 6.83; *sd* = 3.71); and seven young people attended all 24 sessions.

Attendance subgroups were created using two approaches for categorising those who attended a sufficient number of sessions for the intervention to be effective. The treatment provider considered a sufficient dose to be five or more group sessions and six or more one-to-one sessions (i.e. an overall minimum of 11/24 sessions, less than 50%). As a result we look at attendance as a combination of attendance in group and one-to-one sessions. Of the 320 young people, 208 (65%) met these criteria. Analyses were run on these groups in comparison to controls to test the impact of having met the dosage specified by the treatment provider (we refer to this approach as “Provider attendance”).

Other studies have shown that treatment effects may be greater for those who are most compliant and/or those who are exposed to the highest “dosage” of an intervention, providing motivation to examine this further (e.g. [32, 33]). For this reason we also examined whether a group of highest attenders differed in relation to controls. The “High Attenders” group was defined as achieving more than 75% attendance (10–12 sessions) of *both* group and one-to-one sessions. As Table 3 shows only 65 (20%) of young people attended enough sessions to receive this dosage (which we call “LEIP attendance”). A similar cut-off to separate out high-attenders for sub-group analyses has been used by others (e.g. [34]).

**Table 3:** Attendance Breakdown for Young People with Baseline and/or Post-intervention testing (*n* = 320).

	**Group**	**One-to-one**	**Group and one-to-one**
**Provider attendance**	**> 5 sessions *n* (%)**	**>6 sessions *n* (%)**	**> 5 group & >6 one-to-one *n* (%)**
Did not meet criteria	88 (27.5%)	99 (30.9%)	112 (35.0%)
Met criteria	232 (72.5%)	221 (69.1%)	208 (65.0%)
**LEIP Attendance**	**Group**	**One-to-one**	**Group and one-to-one**
No sessions attended	47 (14.7%)	40 (12.5%)	39 (12.2%)
Between 1–9 (< 75%) sessions attended	163 (50.9%)	189 (59.1%)	216 (67.5%)
Between 10–12 (> 75%) sessions attended–“High Attenders”	110 (34.4%)	91 (28.4%)	65 (20.3%)

*Engagement with intervention*: The importance of implementation quality and its impact on the success or failure of interventions has been widely demonstrated [35, 18]. However, *measuring* implementation quality is difficult because it is a multifaceted construct, which includes the quality of programme delivery as well as participant involvement [35, 36]. Measures of programme delivery include: evaluation of adherence to a curriculum; training of staff; time spent on/off task in sessions. Participant involvement can include: consideration of attendance or dosage; participants’ engagement; and behaviour in sessions. Studies investigate different aspects of the overarching concept of implementation quality [35] but use interchangeable terminology. For instance, fidelity is used by some as a synonym for implementation quality (e.g. [36]), yet for others it refers to only one of its components (e.g. see meta-analysis by Durlak & DuPre, [35]).

Mindful of these points, the essence of what we wanted to capture was the extent to which pupils were engaged with sessions. We used data collected in the SPS documents to capture engagement. Those documents set out what was planned in each of the group and one-to-one sessions, and allowed core workers to: (i) score behaviour (compliance) in each session on a 5-point scale ranging from 1 (excellent behaviour, no disruptions) to 5 (very poor behaviour, continuous disruptions); and (ii) rate the amount of time young people spent off/on session task and engaged with the content of the sessions, using a 5-point scale, ranging from 1 (80–100%) to 5 (0–20%). Conceptually this is a mixture of “content covered”, “behaviour” and “perceived engagement” so we treated this as an overall measure of “engagement”. Further, there was a high correlation between the behaviour and time spent on/off task for both the group (*r* = 0.95) and one-to-one sessions (*r* = 0.98), which supports the idea of combining these measures. This is not surprising as the two constructs are closely related; better behaviour allowed more time spent on task and worse behaviour served as distraction from the content of the session, hence was potentially closely relatd to less time spent on task. It is therefore possible that the two questions measured one underlying constructs. Therefore, we combined these scales by averaging them for one-to-one and groups sessions, creating separate “engagement scores” for both group and one-to-one sessions. We then median split these scores to create “low” and “high” engagement groups based on the median of 3.98 (*M* = 3.64; *sd* = 1.18; skewness = - 1.97) for group and 4.73 (*M* = 4.29; *sd* = 1.25; skewness = - 2.76) for one-to-one sessions (see Table 4 for the frequencies). Median split was selected as the most appropriate method to split the sampe based on engagement due to the high negative skew.

**Table 4:** Frequencies for Low and High Group and One-to-one Session Engagement.

	*Young person*		*Teacher*	
	Group	One-to-one	Group	One-to-one
Low (below median), *n* (%)	146 (50%)	145 (48.4%)	140 (48.9%)	145 (49.4%)
High (on or above median), *n* (%)	146 (50%)	154 (51.5%)	146 (51.0%)	148 (50.5%)

**Individual Baseline Characteristics**: We used the baseline self-reports of anti-social behavior, communication, school bond and teacher-child relationships from young people as potential moderators of treatment effects.

*Antisocial behaviour* was assessed by young people completing the pupil version of the Misbehaviour in School (MISQ) measure (devised for the LEIP study; *α* = 0.78). The MISQ captures behaviours most frequently reported as reasons for exclusion in official statistics [37].

*Communication* was assessed by young people completing a 24-item measure, which had previously been used in the pilot evaluation of the programme [6]. This measure was intended to capture pupils’ perception of their communication skills in four areas: understanding, language processing, expressive language, and social communication. The measure comprised questions such as, “can you talk to teachers”, and “can you remember instructions that people tell you?”. Each item was rated on a five-point scale ranging from “Never” to “All the time” (*α* = 0.93).

*School bond and student-teacher relationship*: Three items adapted from the “What’s Happening In this School Questionnaire” (WHSQ; [38]) were utilised for this construct (*α* = 0.72). Young people were also asked four questions adapted from the WHSQ on their relationship with a teacher (*α* = 0.81). Items were rated on a 5-point scale from 1 – Never to 5 – Always.

**Demographic Characteristics**: *Year group and sex*: Year group data as well as information related to sex of the young people were obtained from the schools when young people were nominated for the study and confirmed using data collected from administered surveys. There were *n* = 454 boys and *n* = 191 girls. The mean age of pupils in Year 9 (*n* = 330) and Year 10 (*n* = 314) was 13.01 (*sd* = 0.13), and 13.98 (*sd* = 0.16), respectively.

### Statistical procedure

All analyses were conducted via multilevel logistic regression models where individuals (the level-1 units) were clustered within schools (the level-2 units). Intercepts were allowed to vary by school (i.e. random intercepts were modelled) to account for between-school variability in outcomes. Level-1 predictors were individual-level properties such as sex, adherence to treatment, anti-social behaviour and baseline exclusion (described in more detail below). The only level-2 predictor was treatment allocation.

Models were estimated in *Mplus 6*.*11* [39] using full information maximum likelihood estimation (FIML). FIML estimation provides unbiased parameter estimates provided that data are missing at random (MAR; [40]). Under MAR, the probability of missingness can depend on observed but not unobserved values. Results from complete case analyses (CCA; not shown) were also carried out and did not differ markedly from those reported here. All models were estimated controlling for pupil sex and baseline values of the evaluated outcome. To keep the number of predictors in the model to a minimum, the minimisation variables (listed on page 8) were not included as covariates. In a previous report, presenting main effect analyses of EiE-L, the inclusion of these variables in the model did not have a substantial impact on the estimates of treatment effects [5].

#### Moderation analyses

In order to evaluate whether there was an effect of treatment characteristics—attendance at and engagement in the intervention—we fit the following model for each candidate moderator:

$$Excl_{ij}=B_{0j}+B_{1}BaseExcl_{ij}+\;B_{2}Sex_{ij}+B_{3}Moderator_{ij}+B_{4}\;Moderator\times Treat_{ij}\;+\;e_{ij},$$ (1)

$$B_{0j}=\gamma_{00}+\gamma_{01}Treat_{j}\;+u_{0j},$$ (2)

where *BaseExcl*_*ij*_ is exclusion at baseline for individual *i* in school *j*, *Sex*_*ij*_ is the sex of individual *i* in school *j*; *Moderator*_*ij*_ is the candidate moderator variable for individual *i* in school *j* and *Moderator*_*ij*_ × *Treat*_*ij*_ is a product term formed of the moderator variable and the treatment allocation received by individual *i* in school *j*. In the case of the adherence and attendance variables, *Moderator*_*ij*_ was a dummy coded variable representing whether or an individual met specified criteria for adherence or attendance; in the case of the remaining candidate moderators they were continuous predictors. In either case the candidate moderator was centered to aid interpretation and to avoid non-essential collinearity (e.g. [41]). These variables were centred on the grand rather than group mean, i.e. the overall sample mean for each predictor was subtracted from each predictor value. *β*_1_ is the standardised fixed main effect for exclusion at baseline; *β*_2_ is a standardised fixed main effect of thesex; *β*_3_ is a fixed main effect of the candidate moderator; and *β*_4_ is the fixed moderation effect of the candidate moderator.

In Eq 2, *Treat*_*j*_ is the treatment variable, here a level-2 predictor. In addition, *γ*_01_ is the effect of treatment on school intercept and *u*_0*j*_ is a school-level residual where $u_{0j}\sim N\left(0,\;\sigma_{u}{}^{2}\right)$. The variable ${\tilde{y}}_{ij}$ is a continuous variate underlying the observed outcome variable *Excl*_*ij*_ representing whether individual *i* in school *j* was excluded post-intervention. When ${\tilde{y}}_{ij}$ crosses a threshold = 0, *Excl*_*ij*_ = 1 and otherwise *y*_*ij*_ = 0. The individual-level residual is *ε*_*ij*_ ~logistic(0, $\frac{\pi^{2}}{3}$) i.e. according to the standard logistic distribution.

In line with common practice across the field of psychosocial interventions and the ongoing debate about the need/utility of adjustments in trials with multiple outcomes (e.g. [42]), we did not adjust for multiple outcomes in our estimation of sample size/power or interpretation of findings. The key parameter of interest is *β*_4_, capturing the moderation of treatment by the candidate moderators. The decision to treat *β*_4_ as an individual level effect requires some justification. The model was designed to reflect the research question: do certain properties of the individual (e.g. sex, anti-social behaviour, adherence to the intervention) moderate the impact of treatment on exclusions. The candidate moderators were level-1 predictors and—due to the cluster randomised design of the study—the treatment was a level-2 predictor. Given this design, we judged the optimal statistical test for moderation effects to be to treat the moderation effect i.e. *β*_3_ as a level-1 effect. The two main alternatives were to specify all effects in a single-level model or to specify the moderation effect as a cross-level interaction whereby treatment predicts the slope of the candidate moderators. The former was rejected as a strategy because it does not acknowledge level-2 variability at all. The latter was rejected as a strategy on the basis of a lack of symmetry in cross-level interactions i.e. moderation of individual-level effects on exclusions by treatment is not the same as moderation of treatment effects by individual-level characteristics.

Finally, power to detect moderation is generally low, especially when level-2 units are concerned (e.g. [43, 44]). Any decreases in standard errors and attendant increases in type 1 error rate due to specifying the interaction in this way were, therefore, accepted as providing a reasonable trade-off between power and false positives. When significant moderation effects were identified, we computed simple slopes for the treatment effect at different levels of the moderator. This was done to facilitate interpretation of the nature of the interaction.

## Results

### Intra-class correlation of outcomes

The unconditional baseline intra-class correlations (ICC) for young person reported exclusions was .05 and for teacher-reported exclusions .29. ICCs were calculated in MPlus using the formula presented in Muthén [45].

### Treatment characteristics

With respect to *attendance* (Table 5), compared to controls, there were no significant differences in the likelihood of exclusion for pupils who met (OR = 1.52 for young person and 1.17 for teacher reports) or did not meet (OR = 1.37 and 0.60) the Provider attendance criteria compared to controls. Similarly, there were no significant differences in exclusion found when comparing those who were classified as the “Highest Attenders” and controls (OR = 1.06 and 1.11). However, we did find significant differences between controls and pupils in treatment schools who attended less than 10 of both group and one-to-one sessions (OR = 1.81, *se* = 0.23, *p* = 0.01). Teacher reported exclusions were in the same direction (OR = 1.12), but were not statistically significant. Young people who did not attend any group or one-to-one sessions did not differ in the rate of self-reported exclusions from controls (OR = 0.30). This effect was not modelled for teacher-reported data as no exclusions were reported by teachers in the group of young people who attended no sessions and for whom post-intervention teacher-reported data was available (*n* = 10). Moreover, based on official records of exclusions, no significant differences were found based on attendance.

**Table 5:** Model Results for Attendance.

Treatment characteristic	Provider attendance OR (*se*)	LEIP attendance OR (*se*)
**Young person rated exclusion**		
No sessions	N/A	0.30 (0.81)
Did not meet criteria	1.37 (0.29)	1.81 (0.23)*
Met criteria	1.52 (0.22)	1.06 (0.32)
R^2^ within	0.05 (0.02)*	0.08 (0.04)*
**Teacher rated exclusion**		
No sessions	N/A	N/A
Did not meet criteria	0.60 (0.37)	1.12 (0.36)
Met criteria	1.17 (0.50)	1.11 (0.45)
R^2^ within	0.03 (0.02)	0.528 (0.85)
**Official record of exclusion**		
No sessions	N/A	1.04 (0.70)
Did not meet criteria	1.42 (0.46)	1.60 (0.39)
Met criteria	1.74 (0.38)	1.31 (0.54)
R^2^ within	0.09 (0.05)*	0.09 (0.04)

Note.

* p <. 05;

ORs greater than 1 indicate more likely to be excluded than controls and OR less than 1 indicate less likely to be excluded than controls.

Turning to *treatment engagement* (Table 6), young people in treatment schools were more likely to be excluded compared to controls according to both young person (OR = 1.66, *se* = 0.26, *p* = 0.047) and teacher (OR = 2.31, *se* = 0.37, *p* = 0.023) reports if they attended group sessions with low engagement. Young people who attended groups with high engagement did not differ from controls based on either respondent (OR = 1.50 and 0.65). Low engagement in one-to-one sessions was only related to the odds of being excluded according to the young person reports (OR = 1.97, *se* = 0.26, *p* = 0.009 versus 1.84 for teachers). High engagement in one-to-one sessions, on the other hand, revealed no significant differences based on either respondent (OR = 1.46 and 0.95). Analyses based on the official records of exclusions revealed no significant differences based on group or one-to-one session engagement.

**Table 6:** Model Results for Group and One-to-one Session Engagement.

	Group engagement OR (*se*)	One-to-one engagement OR (*se*)
**Young person rated exclusion**		
Low (below median)	1.66 (0.26)*	1.97 (0.26)**
High (on and above median)	1.50 (0.25)	1.46 (0.24)
R^2^ within	0.05 (0.02)*	0.06 (0.02)*
**Teacher rated exclusion**		
Low (below median)	2.31 (0.37)*	1.84 (0.40)
High (on and above median)	0.65 (0.39)	0.95 (0.39)
R^2^ within	0.06 (0.03)*	0.03 (0.02)
**Official record of exclusion**		
Low (below median)	1.18 (0.46)	1.21 (0.42)
High (on and above median)	1.53 (0.47)	1.48 (0.44)
R^2^ within	0.08 (0.04)	0.08 (0.04)

Note.

* p <. 05;

ORs> 1 indicate more likely to be excluded than controls and OR<1 indicate less likely to be excluded than controls.

### Baseline characteristics

Of the four baseline characteristics assessd as possible moderators of treatment effects only one significantly moderated the effect of the intervention. Specifically, as shown in Table 7, there were no significant interaction effect for allocation by baseline *communication skills* (OR = 0.93 for pupil reports, 1.14 for teacher reports, & 1.97 based on official records), *school bond* (OR = 1.04, 1.26, & 1.88), or *student-teacher relationships* (OR = 1.11, 0.89, 1.93). These results suggest that young people with higher scores (or lower scores) on these characteristics were equally unlikely to benefit from the intervention based on young person and teacher reports, as well as official records. However, baseline *antisocial behaviour* was a significant moderator of the effect of the intervention effect on teacher-reported school exclusion (OR = 0.38, *se* = 0.38, *p* = 0.012; R^2^ = 0.06; *p* = 0.037). To characterise the nature of this interaction, we computed the simple slopes for the intervention effect at different levels of baseline anti-social behaviour. We computed treatment effect at 1SD below the mean on baseline antisocial behaviour, at the mean of antisocial behaviour and 1SD above the mean of antisocial behaviour. The odds ratios for the treatment effect at 1SD below the mean of baseline antisocial behaviour was 1.75; at the mean it was 0.96; and at 1SD above the mean it was 0.52. Thus, pupils in the treatment group with worse baseline behaviour were reported by their teachers to receive fewer exclusions than pupils with the same behaviours in the control group. On the other hand pupils in the treatment group with better baseline behaviour were reported by their teachers to receive more exclusions than pupils with comparable baseline behaviour in the control group. This moderation effect was not significant based on young person reported exclusions or official records of exclusion.

**Table 7:** Estimates for Baseline and Demographic Moderator/Characteristics.

	Anti-social behaviour	Communication	School bond	Teacher-student relationship	Sex	Year group
	OR (*se*)	OR (*se*)	OR (*se*)	OR (*se*)	OR (*se*)	OR (*se*)
	***Young person rated exclusion***					
Baseline Exclusion	1.86 (0.20)**	1.99 (0.19) ***	1.87 (0.20)**	2.02 (0.20)***	2.0 (0.20)***	2.07 (0.20)***
Sex	0.85 (0.21)	0.83 (0.21)	0.78 (0.22)	0.85 (0.21)	N/A	0.85 (0.22)
Allocation	1.49 (0.21)	1.43 (0.20)	1.48 (0.21)	1.42 (0.21)	1.49 (0.22)	1.45 (0.22)
Moderator main effect	1.30 (0.16)	0.82 (0.13)	0.68 (0.10)***	0.81 (0.10)*	0.82 (0.22)	0.95 (0.19)
Moderator interaction effect	0.74 (0.31)	0.93 (0.26)	1.04 (0.20)	1.11 (0.21)	1.80 (0.43)	0.53 (0.38)
R^2^ within	0.04 (0.02)*	0.04 (0.02)*	0.07 (0.03) **	0.05 (0.02)*	0.04 (0.02)*	0.05 (0.02)*
R^2^ between	0.34 (0.34)	0.38 (0.36)	0.32 (0.31)	0.35 (0.36)	0.37 (0.40)	0.33 (0.39)
	***Teacher rated exclusion***					
Baseline Exclusion	1.50 (0.26)	1.55 (0.26)	1.51 (0.26)	1.55 (0.26)	1.52 (0.26)	1.62 (0.27)
Sex	1.17 (0.29)	1.85 (0.29)	1.13 (0.30)	1.92 (0.29)	N/A	1.12 (0.29)
Allocation	0.96 (0.36)	1.02 (0.37)	1.02 (0.38)	1.04 (0.37)	1.04 (0.29)	1.03 (0.37)
Moderator main effect	1.64 (0.19)**	0.95 (0.17)	0.87 (0.13)	0.84 (0.13)	1.25 (0.38)	0.63 (0.24)*
Moderator interaction effect	0.38 (0.38)*	1.14 (0.34)	1.26 (0.25)	0.89 (0.26)	1.73 (0.58)	0.25 (0.48)**
R^2^ within	0.06 (0.03)*	0.02 (0.02)	0.02 (0.02)	0.02 (0.02)	0.02 (0.02)	0.07 (0.03)*
R^2^ between	0.005 (0.03)	0.00 (0.01)	0.00 (0.003)	0.001 (0.01)	0.001 (0.01)	0.004 (0.03)
	***Official record of exclusion***					
Baseline Exclusion	2.79 (0.30)	2.78 (0.30)	2.82(0.30)	2.80 (0.30)	2.76 (0.30)	2.87 (0.30)
Sex	1.36 (0.41)	1.36 (0.40)	1.26 (0.40)	1.41 (0.39)	N/A	1.32 (0.40)
Allocation	1.57 (0.41)	1.60 (0.41)	1.70 (0.43)	1.72 (0.42)	1.49 (0.40)	1.68 (0.43)
Moderator main effect	1.04 (0.29)	0.90 (0.23)	0.78 (0.19)	0.73 (0.19)	1.25 (0.40)	0.56 (0.31)
Moderator interaction effect	0.82 (0.58)	1.97 (0.46)	1.88 (0.38)	1.93 (0.37)	2.66 (0.78)	1.62 (0.62)
R^2^ within	0.07 (0.04)	0.09 (0.05)	0.11 (0.05)*	0.12 (0.05)*	0.08 (0.04)	0.10 (0.05)*
R^2^ between	0.10 (0.16)	0.10 (0.16)	0.12 (0.17)	0.13 (0.18)	0.11 (0.19)	0.12 (0.17)

Note.

* p < .05;

** *p*< .01;

*** *p* < .001;

Main and moderator effects are controlling for exclusions at baseline and sex; under “allocation” we provide estimates for the main effect of allocation; under “moderator main effect” we provide estimates for the main effect of the moderator variable; and under “moderator interaction effect” we provide estimates for the allocation by moderator interaction.

### Demographic characteristics

Table 7 also contains results for demographic characteristics. No significant effects were observed for the differential impact of the intervention on *girls versus boys* (OR = 1.80 for pupil, 1.73 for teacher reports, & 2.66 based on official records). However, significant differences were observed with respect to *year group* based on teacher-reported exclusions (OR = 0.25, *se* = 0.48, *p* = 0.004). The OR associated with the intervention was 1.80 in year 9 pupils and 0.45 in year 10 pupils. Thus, the nature of the interaction was that the treatment was associated with more teacher reported exclusions in year 9 pupils but a fewer teacher reported exclusions in year 10 pupils compared to controls. This moderation effect was not significant based on young person reported exclusions or official records of exclusion.

#### Supplementary analysis of attendance sub-groups

As differences in outcome emerged related to different levels of attendance we carried out supplementary analyses of the sample on measures of individual characteristics taken from baseline testing. Young people who attended 1–18 sessions were compared with young people who attended 19–24 sessions revealing that those who attended between 1–18 sessions self-reported higher rates of substance use but were also reported by teachers to have more difficulties in their communication.

## Discussion

Examination of the main effects of the EiE-L intervention revealed negative and null findings in relation to the primary outcome and a set of secondary outcomes [5]. In this paper, we carried out sub-group analyses to examine a set of pre-specified characteristics as potential moderators of the effects of EiE-L on the primary outcome—school exclusion. These analyses revealed few significant moderator effects. Negative effects were found in relation to treatment characteristics. Specifically, lower attendance and engagement with the intervention was related to negative outcomes. Only one of the four baseline characteristics revealed a significant moderation effect, with different baseline levels of antisocial behaviour leading to differential responses to treatment. Finally, demographic characteristics revealed a significant moderation effect relating to the year group of the young people but not in relation to the young people’s sex.

The analyses of treatment characteristics revealed the greatest number of significant moderator effects. Young people who attended up to 75% of the total sessions were more likely to report exclusions than controls, in contrast to non-attenders and High Attenders, who were no different to controls. This finding was supported by effects in the same direction on teacher-reported data and official records, although these results were not statistically significant. Low engagement in group and one-to-one sessions led to young people self-reporting more exclusions than controls. This was supported by teacher-reported data for those attending group sessions with low engagement. Again, teacher-reported data for one-to-one sessions showed the same direction of effect, as did official records for both group and one-to-one sessions, although these supporting findings were not statistically significant. As with our findings related to attendance, young people who attended sessions with high engagement were no different to controls.

Not being able to demonstrate a positive relationship for high attenders versus controls is strongly indicative of the ineffectiveness of the intervention, but also captures possible self-selection within the treatment condition. Supplementary analyses suggest that High Attenders (19–24 sessions) had lower levels of substance use and better communication when compared to lower attenders (1–18 sessions). Hence, High Attenders may have been qualitatively different from those who attended fewer sessions. However, as noted, even these young people failed to benefit from the intervention. This means that when isolating the seemingly less problematic young people in the study, who achieved high attendance, the intervention still failed to make a positive impact. However, we believe it would be wrong to consider this purely as a selection problem. The intervention was premised on engaging young people—even as far as including “Engage” in the name of the intervention- yet achieving good attendance and engagement with the programme appeared to have been problematic for the providers. Furthermore, when good levels of engagement were not achieved, young people reported worse outcomes as young people with low engagement scores in group sessions self-reported more exclusions than controls. The complementary nature of the findings from different data sources points to possible fidelity/implementation problems [36].

Deviancy training has been proposed by several authors as an explanation for iatrogenic effects found in group-based interventions (e.g. [46, 47]). Deviancy training happens when peers reinforce and reward deviant behaviour and create new group norms [47]. Whereas researchers have often relied solely on group session attendance to test for the possibility of deviancy training, we utilised a measure of engagement which considered the behaviour in the group sessions and the amount of time devoted to the planned curriculum. Therefore a low score on our group engagement measure could be taken to indicate a certain lack of structure in those sessions. As group sessions typically contained up to twelve of the highest-risk pupils, it is reasonable to imagine that sessions could become unstructured. If this was the case, then it is known that unstructured group sessions are related to deviancy training/peer contagion [48].

Whilst peer contagion in a group intervention setting appears plausible, there is little high quality research establishing this as a reliable explanation [49] and the estimation of peer effects is inherently difficult [50, 51]. Given that deviancy training is still contested and our findings are inconclusive, we are reluctant to commit to this interpretation as the primary cause for negative effects. We believe it more plausible that if deviancy training occurred it is likely to be due to problems with the intervention programme content and delivery and therefore as much a symptom of other problems as a cause of them.

Further support for favouring implementation problems as a primary explanation for negative effects are found in our results related to engagement in one-to-one sessions. Young people who attended one-to-one sessions with lower engagement self-reported more exclusions than controls. Bearing in mind that core workers only had to monitor and keep engaged one young person rather than up to twelve, this finding speaks more to the session content and the ability of the core workers to engage pupils in activities. Other studies such as Karcher [52] found negative effects after participation in school-based mentoring programmes that were similar in format to EiE-L one-to-one sessions. It is therefore possible that the format of one-to-one sessions led to negative results for those young people who were less engaged. However, these two possibilities are not mutually exclusive as design and implementation problems are often linked [33].

Problems with attendance and engagement are perhaps more likely when dealing with a high-risk sample. However, the intervention provider appears to have had low expectations for the attendance and engagement of young people, despite aiming to alter their behaviour. For example, the attendance criteria they believed to be sufficient seemed low and showed no significant differences when examined in relation to reported exclusions. Furthermore, the intervention design allowed for home visits and telephone calls to the young people’s families, which could have been employed to address attendance and engagement problems. However, comparatively few phone calls were made (*n* = 164) and only eleven home visits were completed. For illustration purposes, 47 young people did not attend any group sessions at all. If one phone call had been made for each session that these 47 young people alone did not attend, then a total of 564 phone calls would have been made. This seems like a missed opportunity for re-engaging youths and their families in the programme and in their education more generally.

Of the four assessed baseline characteristics, three revealed no significant moderation effects. Baseline communication levels did not affect response to treatment, despite the intervention focusing specifically on improving these skills. Previous research suggested that pre-existing communication skills may influence participant’s response to treatment (e.g. [22]). However, it is possible that deficits in these skills may not be directly related to behaviours that result in exclusion, as young people did not achieve better outcomes if they had greater communication skills. Extensive research also suggested that student-teacher relationships are important for young people’s long-term welfare at school ([24]). However, our findings did not provide evidence that these relationships lead to positive responses to intervention programmes. Similarly, the young people’s bond to their school revealed no difference in outcomes following the intervention. Although we attempted to measure an aspect of how the young people in this study felt about their school and teachers, we did so on an individual basis. This may have failed to demonstrate differences in response to treatment due to measuring a school-level characteristic on an individual basis. Attempting to measure a school *culture*, rather than an individual perception, may provide insight into the role of schools and school climate in managing behaviour and high-risk young people. Other research has shown that school climate can be manipulated to provide a nurturing and supportive environment in which targeted assistance can be provided alongside school-wide initiatives [53, 54]. research exploring the role of current school climates may demonstrate the benefit of such an approach. Student-teacher relationships and connectedness to school should be explored in future studies to further understand the potential of environment and relationships in improving the school experience of troubled young people, especially as excludees recognise their importance [28].

Previous research suggested that differences in response to treatment might be observed according to differences in levels of pre-existing behaviour [20]. The effect of baseline levels of antisocial behaviour differed for young people in the treatment and control groups. In our study, perhaps counter-intuitively, pupils in the treatment group with worse baseline behaviour were reported by their teachers to receive less exclusions than pupils with the same behaviours in the control group. On the other hand, pupils with better baseline behaviour who received the intervention were reported to receive more exclusions than their counterparts in the control schools. It is possible that less antisocial young people were influenced by those with greater antisocial behaviour, resulting in a worsening of behaviour and thus increased exclusions. This may be a result and further support fora deviancy training effect, as discussed above. That young people with more antisocial behaviour are excluded less is more difficult to understand. They may have been positively affected by the intervention, by associating with less deviant peers, or by benefiting from the material taught in group and one-to-one sessions. Alternatively, as this finding is based on teacher-reports, the young people may have been viewed differently by teachers due to their enrolment on the programme. For instance, teachers may have had less contact time with the young people and so viewed them differently. It is also possible that teacher questionnaires were inadequately completed. The higher degree of variability in teacher reporting than in young person reports as demonstrated by a higher ICC may suggest less reliability for this data and results. However, we do not know what effect the additional variability has beyond making the identification of statistically significant differences more difficult. Therefore, one might view the additional variability between teachers as some indication that data were not reliable in the first place, as teacher reports were very different between schools. It should be noted that the same direction of effect is observed, although this is statistically non-significant in findings from the young person questionnaire and official records. This perhaps limits the possibility that the variability in the teacher questionnaires is solely responsible for this finding and future studies should focus on examining these effects further.

Finally, analyses of demographic characteristics showed significant differences in response to treatment for young people depending on their year group. However, there was no moderator effect for sex, in spite of the large differences in the likelihood of exclusion for males and females [9]. This may be a reflection of the nature of the sample, in that generally observed sex differences may be less significant in a sample of high-risk youths [54]. The findings for pupils’ year group were both positive and negative. Teachers of Year 9 pupils in treatment schools were more likely to report exclusions than controls; whereas teachers of Year 10 pupils were less likely to report exclusion. The interaction was not significant for the young person reported exclusions. While the positive Year 10 results may seem encouraging, it is tempered by the fact that we did not observe statistically significant reductions in exclusions according to self-reports. Furthermore, results for this moderator derived from official records showed the opposite direction of effect, meaning that we can be less confident about this finding. It is therefore possible that these as the findings for baseline antisocial behaviour may reflect the additional variability in the teacher reported data, meaning it must be interpreted with caution and further research is warranted.

Our study has a number of strengths. We have added to the limited knowledge base on school-based interventions for high-risk young people. Furthermore, this study begins to address the paucity of studies that report moderator analyses, despite an acknowledged need for such work [55]. The trial also took place in real-world settings meaning that we can be more confident about the likely portability of such an approach to similar settings (i.e. schools in deprived areas). Further, we attempted to measure aspects of the intervention and young people that other research suggested were important in achieving intervention effects. To this end we analysed two components of implementation quality and considered a wide range of baseline characteristics. We also used multiple informants and official records in order to cross-validate results and our significant findings are supported by patterns in the same direction across informants.

However, this study also has limitations. Whilst the trial was powered for the main effects analysis, the sample size is relatively small for assessing moderator effects and sub-group analyses more generally [56]. This means that the results of the follow-up analyses, in particular, should be viewed as exploratory in nature [57]. But given the dearth of such studies, such exploratory analyses are still valuable. As stated, the teacher reported data may present measurement problems which impact on the reliability of a small number of findings. However, with the exception of one, the findings are replicated based on young person reports.

Researchers have pointed to difficulties related to subgroup analyses that can impact on the credibility of findings [2, 56, 57]. The difficulties include “data-dredging” approaches, inadequate modelling and insufficient statistical power to detect effects in smaller samples. These types of problems may lead to inaccuracies when interpreting sub-group results. However, authors also agree that sub-group analyses provide valuable in depth understanding of the treatment and its effects, positive or negative, on groups of individuals. As the benefits outweigh the potential negatives, efforts should be made to conduct subgroup analyses but do so in a rigorous manner. To this end, approaches have been suggested which can maximise the credibility of subgroup analyses and minimise potential false findings [2, 57]. In the current project we followed these guidelines as fully as possible. Subgroups were pre-specified in the protocol and hypotheses were stated when existing literature provided reasonable evidence that a certain effect could be expected. These subgroups were analysed in relation to the primary outcome only, and testing for interaction effects was conducted where possible [2].

## Conclusion

Overall, our findings support the main effect analyses in that the intervention was not successful in achieving positive change. Sub-group analyses revealed predominantly null results, but showed that some young people responded negatively to the intervention. Consistent with Zane et al. [3, 33], our analyses revealed a range of possible interrelated reasons underlying the negative and null effects of EiE-L. A combination of design and implementation problems, possibly compounded by deviancy training, appears to best explain our sub-group findings. As such, they also offer insights into the understanding of the main effect results [5]. These findings imply that a high level of implementation is crucial to deliver an effective intervention [58] or avoid negative effects [3]. That some young people were not sufficiently engaged and went on to report worse outcomes than controls suggests that working ineffectively with high-risk young people may actually be worse than doing nothing at all. It should be sobering for both practitioners and policy-makers who feel compelled to “do something”, particularly when it comes to children and young people. When considering what *can* be done, there is a growing body of evidence about effective school-wide interventions that do not pathologise individual pupils and that work to improve the overall school climate [54].

## Supporting Information

S1 CONSORT Checklist Consort Checklist. (PDF)

S1 Protocol Article published in *BMC Psychology*, 2:24. (PDF)

S1 Text LEIP Moderation Power Analysis and Table A. (PDF)
