# Supplementary material for: London Education and Inclusion Project (LEIP): Exploring Negative and Null Effects of a Cluster-Randomised School-Intervention to Reduce School Exclusion—Findings from Protocol-Based Subgroup Analyses
Source: PLoS One. 2016 Apr 5;11(4):e0152423. doi: 10.1371/journal.pone.0152423 (PMC4821581; doi:10.1371/journal.pone.0152423)
Supplement: S1 Protocol — (PDF) [file pone.0152423.s002.pdf]

BMC Psychology

STUDY PROTOCOL Open Access

London Education and Inclusion Project (LEIP): A cluster-randomised controlled trial protocol of an intervention to reduce antisocial behaviour and improve educational/occupational attainment for pupils at risk of school exclusion

Ingrid Obsuth[1*], Alex Sutherland[1], Liv Pilbeam[1], Sarah Scott[1], Sara Valdebenito[1], Rosanna Carr[2] and Manuel Eisner[1]

Abstract

Background: In 2011/12 about 6% of pupils in England who were in the last two years of compulsory education (Years 10 and 11) experienced one or more fixed period school exclusions[a] for disciplinary reasons and there are roughly 300,000 fixed period exclusions every year in England and Wales (Department for Education, 2013a). Excluded pupils are at a greatly increased risk of failing GCSE examinations, not being in employment, education or training (NEET) at ages 16–24, and having criminal convictions as adolescents or young adults. To date, little or no research has been conducted on programmes designed to improve outcomes for those at risk for fixed period exclusions. Similarly, there is very little research on the effects of school disciplinary procedures, such as fixed period exclusions, on outcomes for young people.

Method/Design: The current study attempts to fill these gaps via a cluster-randomised controlled field experiment designed to evaluate the effectiveness of a social and communication skills based intervention for Year 9 and 10 pupils at high risk for fixed-term exclusion during the 2013/14 academic year in selected Greater London schools. The project will chart the short-, medium- and long-term effects of the intervention on the participants, as well as track the participants via administrative records over time.

Discussion: It is an independent evaluation, in which the role of the evaluation and the programme implementation are separated and carried out by two independent teams funded by different agencies.

Trial registration: Current Controlled Trials: ISRCTN23244695 (14 Jan 2014).

Keywords: Fixed-term school exclusion, High-risk adolescents, Disciplinary procedures, Schools

Background

What is exclusion?

The 2002 Education Act governs the use of school exclusion as a disciplinary measure and defines two types of exclusion: permanent and fixed term.[b] Permanent exclusion means that a pupil is permanently removed from a given school whereas fixed term exclusion lasts between one and a maximum of 45 days per school year (i.e., nearly 25% of a 39 week school year; for more details, see: Centre for Social Justice, 2011). There were 304,370 fixed period exclusions across all maintained primary, state-funded secondary and special needs schools in 2011/12, equating to 4.05% of the school population being given a fixed term exclusion at least once during that school year (Department for Education, 2013a). Most school exclusions occur during secondary school, between ages 11 and 16. The rate of exclusion peaks during the last three years of compulsory school (i.e., Years 9–11). Amongst these cohorts, 7.8% of male

* Correspondence: io229@cam.ac.uk
[1]Institute of Criminology, University of Cambridge, Sidgwick Avenue, Cambridge CB3 9DA, UK
Full list of author information is available at the end of the article

BioMed Central

© 2014 Obsuth et al.; licensee BioMed Central Ltd. This is an Open Access article distributed under the terms of the Creative Commons Attribution License (http://creativecommons.org/licenses/by/4.0), which permits unrestricted use, distribution, and reproduction in any medium, provided the original work is properly credited. The Creative Commons Public Domain Dedication waiver (http://creativecommons.org/publicdomain/zero/1.0/) applies to the data made available in this article, unless otherwise stated.

pupils and 3.6% of female pupils experience exclusion at least once per school year.

If a pupil is subject to a fixed term exclusion of six days or more, schools must provide alternative full-time education (the so-called 'six-day rule'). Headteachers arrange a reintegration interview with the parents of pupils excluded at primary school and for pupils excluded for more than five days at secondary schools. However, schools are only required to provide homework if a pupil is excluded for less than six days (Department for Education, 2013b).

Who is excluded?

Male pupils, children from deprived and (some) ethnic minority backgrounds are much more likely to be excluded than their counterparts (see Department for Education, 2013a).[c] In particular, children with special educational needs (SEN) experience rates of exclusion far higher than their counterparts. For example, around 11% of SEN pupils were temporarily excluded from secondary schools in 2011/12. By comparison, only 2.55% of students *without* SEN experienced school exclusion (Department for Education, 2013a). Meltzer (2003) also found that the rate of exclusion is significantly higher (between 10–25 times the prevalence in other groups) for children with diagnosed conduct/hyperkinetic disorders or mental health problems. Excluded children are also often at an early disadvantage as many are found to have educational difficulties that were not identified or adequately addressed earlier (Macrae et al. 2003). In addition, up to 66% of excluded children are reported to have communication difficulties, identified or not by their schools (Clegg et al. 2009). Excluded children are also disproportionately likely to come from lone-parent families, families where parents have educational difficulties of their own, or have stressful home environments in general (Macrae et al. 2003; Munn et al. 2000). To summarise, the demographic and socioeconomic patterns of who is excluded do not appear to have changed substantially: those who are poor; males; from ethnic minority backgrounds; with pre-existing physical, social, or psychological difficulties, or educational needs; are typically those who are excluded from schools in England.

Why are children excluded?

Government data show that three-quarters of fixed term exclusions in the UK are for aggressive externalising behaviour.[d] Most (recorded) exclusions appear to be a direct and routine response to aggressive or disruptive behaviour, but schools retain considerable discretion with regard to the length of exclusion and whether to exclude or not. Macrae and colleagues (2003) point to several key factors that contribute to the decision to exclude, including the disciplinary policies and the level of tolerance of the headteachers in individual schools. We know that, for example, rules and enforcement regarding school uniform varies between schools. Whilst school uniforms are strongly encouraged by the Department for Education, there is no general, nation-wide legislation regulating their implementation or endorsement (Department for Education, 2013c). As such, school *policies* should also be included in a discussion about reasons why children are excluded (see Galloway et al. 1985; Hayden 2009). A more ephemeral institutional factor, which features heavily in discussions about the possible criminogenic effects of school exclusion and the extent to which 'school effects' exist, is school *ethos* (see Rutter et al. 1979; Boxford 2006)[e].

What effect(s) does exclusion have?

In the short-term and medium-term, school exclusion is correlated with several behavioural and educational problems. For the young person, school exclusion has been found to be related to poor academic and occupational outcomes, externalizing behaviour including crime and negative internalizing outcomes, such as self-harm (Massey 2011; Sparkes 1999; Graham 1988; McAra and McVie 2010). Furthermore, Gilbertson (1998) showed that 42% of sentenced juvenile offenders had experienced, a previous school exclusion. In the long-term, school exclusion is correlated with later unemployment.[f] Speilhofer (2009) showed that amongst those young people who were long-term NEET (Not in Education, Employment or Training) the majority have previous exclusions and truancy. A recent study also suggests that approximately 50% of excluded children become NEET within two years after their exclusion (Massey 2011). Taken together these data suggest that children who are subject to temporary or permanent school exclusion are at a much greater risk of behavioural, health-related, occupational and educational difficulties.

However, it is important to point out that while these studies are very convincing in supporting a strong link between school exclusions and adverse outcomes prospectively and retrospectively, they do not address the issue of a causal relation in this link. In other words, from the evidence thus far, it is not clear whether school exclusion is simply a marker or a causal factor in subsequent negative development. In fact, it is possible, that school exclusions as well as the commonly assessed adverse 'outcomes' are both the consequence of a common third factor or factors, for example, a personality characteristic of the young person, combined with characteristics of the family, school, or particular policy. To address the question of causality one would want to carry out an experiment, in which young people would be randomly assigned to being excluded or not. In this way, school exclusion would be the

only systematic difference between the two groups, thus any subsequent difference between the groups could be attributed to school exclusion. However, for ethical reasons this is not an experiment one can carry out. For situations such as these, where random assignment is not easily achievable, researchers (e.g., Jaffee et al. 2012) have called for differentiating causal links utilizing propensity score matching PSM (Rosenbaum and Rubin 1983; Rosenbaum and Rubin 1985).

The negative effects of a sanction, such as school exclusion may be causally linked to negative outcomes through one or more of the following processes. In line with defiance theory (e.g., Sherman 1993) children who are excluded may escalate their engagement in the negative behaviours that led to the exclusion if they a) perceive this sanction as unfair, b) have a poor school bond, c) feel stigmatized by being excluded, and d) feel no, or deny feeling, shame about being excluded. It is also possible that by being labelled as a 'bad guy', young people identify themselves with this label and through the process of self-fulfilling prophecy (Rosenthal and Jacobsen 1968) engage and escalate in behaviours that originally lead to this label. Alternatively, in accordance with crime opportunity theory (e.g., Cohen et al. 1980) by being excluded from school, an adaptive social environment, young people may have more opportunities to spend time in less adaptive social environments, which may in turn offer increased opportunities to engage in antisocial activities. These are just a few plausible mechanisms linking school exclusion to negative outcomes. However, as mentioned above very little is known about this causal link or its mechanisms, thus warranting further exploration.

What we know thus far is that young people who are excluded tend to be 'hard to reach', disruptive and in many cases aggressive towards adults and/or other pupils, as the statistics above attest. They often have communication difficulties, which may compromise their ability to benefit from the curriculum as well as behave in prosocial ways. Further, children who have experienced exclusion sometimes carry with them the burden of difficulties their parents had with school, or come from home environments that are far from conducive to educational attainment (or more basically, have problems training young children how to behave). Yet in spite of these issues, many thousands of children, who already have a constellation of risk factors for a range of negative life outcomes, are (sometimes repeatedly) exposed to yet another risk factor by being excluded from school. The irony being that those excluded may not like school in the first place, perhaps partly as a result of finding school difficult due to their educational needs. Indeed previous research has shown that children view exclusions as akin to school sanctioned holidays (Dupper et al. 2009). A risk is also that exclusion could weaken (perhaps already fragile) commitment to school that some children have through removing the fear of punishment. Furthermore, it is the most explicit form of rejection by the educational system (Munn and Lloyd 2005).

In summary, pupils experiencing fixed-term exclusions in the UK generally receive minimal support despite exclusion being a risk factor for numerous negative life outcomes. The goals of this study are two-fold; to assess the efficacy of a new intervention targeted at those most at risk for exclusion and to begin to elucidate some of the processes through which school exclusion may be related to adverse outcomes. The evaluated intervention aims to develop the young peoples' communication and broader social skills in order to facilitate more adaptive interactions (prosocial behaviours) with others and eliminate problem behaviours often linked to school exclusion.

Research Plan: impact evaluation

Research questions

This project has several research questions relating to the different outcomes being assessed. Does the intervention affect the:

1. Behaviour of participants in terms of officially recorded truancy, temporary and/or permanent exclusions?
2. Self- or teacher-reported disruptive behaviour of participants?
3. Educational attainment of participants in terms of GCSE or other formal tests (e.g., SATs)?
4. Communication skills of participants in terms of their expressive language, understanding, language processing, and/or social communication skills?
5. Self-reported and officially recorded delinquent and/or criminal behaviour of participants?
6. Likelihood of being Not in Education Employment or Training (NEET) once the children complete compulsory schooling?

Methods/Design

Sample/Participants

School identification and recruitment

In May 2013, all secondary schools in Inner London with a free-school meal (FSM) rate equal to or greater than or equal to 28% were invited to participate in the study (n = 108)[g]. This list excluded specialist schools for physical, emotional or behavioural difficulties such as Pupil Referral Units or so-called 'special' schools. This list also excluded schools (n = 40) that were already participating in initiatives funded by the European Social Fund (ESF) and the Greater London Authority (GLA) aimed at similar groups of young people. Schools were ranked according to the proportion of students with English as another

language (EAL), special educational needs (SEN) and the unauthorised absence rate (truancy). We initially approached schools via letter, detailing the study and invitation to participate in the study, following up via email and telephone. Interested schools were invited to send back to us an Expression of Interest (EOI) document, which was followed up via telephone. Initial progress with recruitment was slow. To ensure that enough schools/pupils are recruited to ensure minimum statistical power (see section below), a second phase of school recruitment took place in a small number of Outer London boroughs on the basis of (1) the school having a FSM prevalence > =28% (2) the number of schools in a given borough; and (3) physical proximity to schools already in the study. Interested schools were invited to an Information Event, during which the study was further explained to them. At the end of recruitment 29 of the 36 schools included in the study were present at the initial Information Event.

Pupil identification and recruitment

The target groups were Year 9 and 10 pupils at high risk for fixed-term exclusion ('suspension') from school during the 2013/14 academic year in select schools in Greater London. The planned intervention is intended for children in the top 3-5% of a school's Year 9/10 populations in terms of problematic behaviour. Within each school, 16–24 young people (based on school size) at the highest risk for fixed term exclusion in Years 9 and 10 were selected for participation (8–12 in each year) by the schools. The planned sample size for the study was 350–400 participants in each arm of the trial with a projected total of 750–800 young people. Prior to randomisation, schools were asked to identify between 10–12 pupils per year who are at greatest risk for exclusion, with a view to having groups of a maximum of 12 per school/intervention.

The guidelines asked schools to select the young people who are at high risk for school exclusion and/or becoming NEET based on a) having had previous school exclusions, b) unauthorized absences, and c) having engaged in behaviours that lead to other disciplinary measures previously being used.

Setting

The study is conducted in each of the participating schools located throughout the following London boroughs: Hammersmith and Fulham (5 schools), Ealing (5 schools), Newham (4 schools), Haringey (3 schools), Tower Hamlets (2 Schools), Barking and Dagenham (3 schools), Kensington and Chelsea (3 schools), Southwark (2 schools), Camden (3 schools), Islington (1 schools), Westminster (2 schools), Waltham Forest (1 school), Wandsworth (1 school) and Lambeth (1 school) (see Figure 1 CONSORT flowchart – school recruitment and randomisation).

The intervention

The intervention was selected through a bidding process organised by the Education Endowment Foundation (EEF),[h] the funding body for the intervention component of the current project. Following a call for proposals from organisations that had an evidence-based approach to working with 14–16 year old pupils at risk of exclusion in London, the EEF received 20 applications. The EEF shortlisted five applicants in line with their mission statement,[i] evidence of impact, scalability and willingness to be evaluated as part of an RCT. Of the shortlisted interventions, the evaluation team selected the Engage in Education London (EiEL) programme (described below), which provided the clearest description of aims, most convincing mechanisms of change and promising findings from a preliminary evaluation Catch22 (Catch22 2013a).

The selected intervention is a 12-week-long programme targeting young people's communication and broader social skills. It consists of weekly group and one-to-one sessions. The intervention is delivered by Catch22, a social business providing services to people in difficult situations, in close collaboration with I CAN, the children's communication charity. Catch22 has a history of working with troubled and vulnerable individuals, with the goal to steer them clear of crime or substance abuse and toward educational and employment attainment (Catch22 2013a). EiEL is a shorter version of the Engage in Education (EiE) programme[j] offered by Catch22 since 2011 throughout the UK. The EiE programme underwent an initial evaluation by the Department for Education (Catch22 2013b). In this pilot study researchers found promising effects in a pre-post design with 1,693 participants. The findings suggested positive effects on a variety of outcomes including school attendance, attainment, and problem behaviour. For example, the report suggested that fixed period exclusions had decreased by 21% following EiE (Catch22 2013a). While the lack of a control group limited the extent to which causal inference could be drawn, the positive changes across a range of outcomes were deemed encouraging.

EiEL was adapted specifically for this group of young people by Catch22 and I CAN, who were involved in the development of the original intervention. The current programme was adapted to be specifically delivered to schools/academies within the LEIP project, with the goal to increase the attendance and attainment of pupils most at risk of fixed-term or permanent exclusion. Following the EiE initial evaluation, EiE staff was consulted when developing the adaptation for the London Education and Inclusion Project (LEIP). The EiEL programme

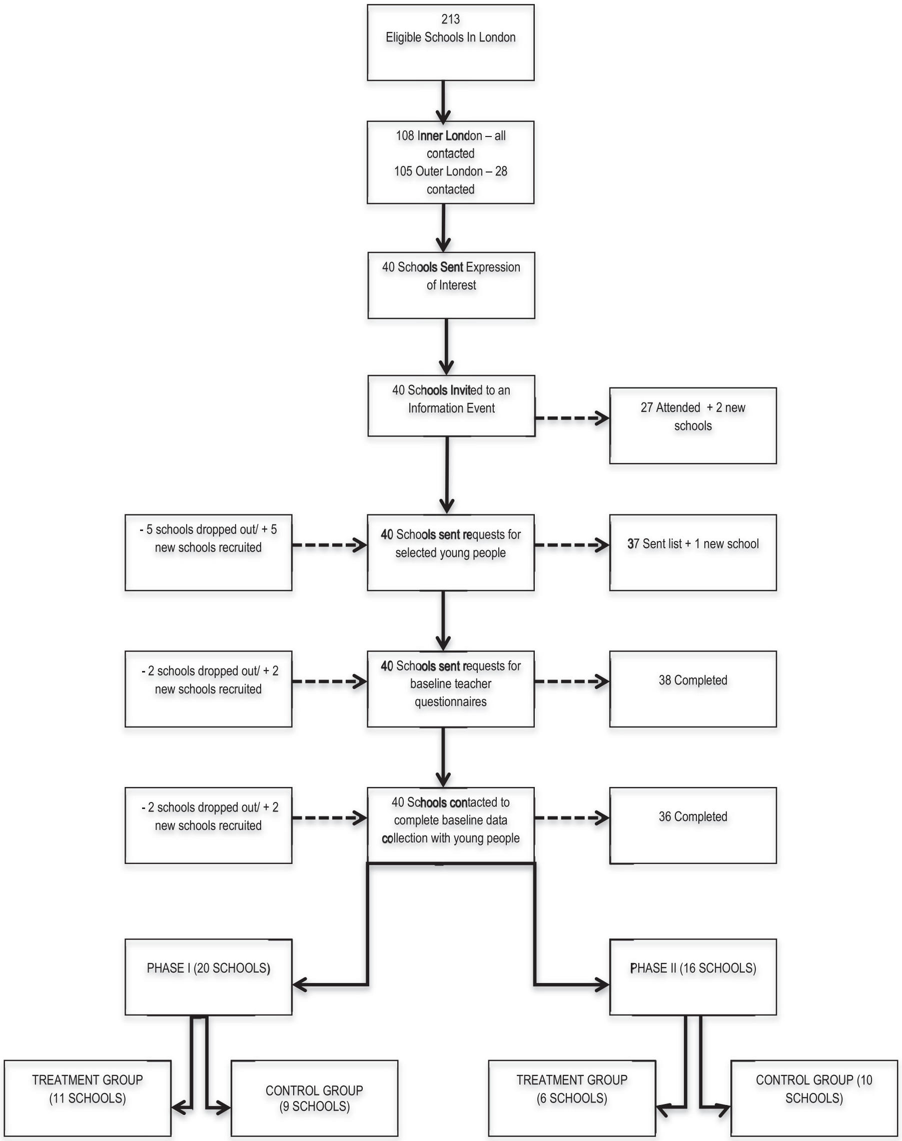

Figure 1 CONSORT flowchart – school recruitment and randomisation.

continues with the EiE intervention approach of each young person attending a weekly group and one-to-one session but the resources have been adapted to fit a shorter 12-week delivery period. The 12-week scheme of work was developed based on a review and identification of activities/strategies, which were found most effective in the initial evaluation.

The intervention targets a number of individual risk factors including: students' communication skills (e.g., ineffective strategies to solve problems, difficulties retelling events, poor conversation skills, difficulties sharing emotions, and understanding the link between cause and effect); hidden communication needs (e.g., receptive-expressive language difficulties); behavioural problems in school (e.g., disruptive behaviour in the classrooms, violence); academic problems, poor attainment and attendance below the expected level. At the family level, the intervention targets risk factors such as poor family support for academic activities whereas at the school level, the intervention is focused on risk factors such as poor classroom management (Ellis 2013).

One of the basic assumptions grounding the intervention is that communication difficulties play a role in behavioural problems at school. Put another way, children who are unable to understand instructions, negotiate in an assertive manner or require further explanations, and may display maladaptive behaviours such as, social withdrawal, somatic complains or aggressive behaviours (see Carr and Durand 1985; Clegg et al. 2009; Van Daal et al. 2007). In addition, the intervention builds on the assumption that the social environment plays an important role in young

people's development. Catch22 contend that positive change is achievable when family members, teachers, and other members of the school environment are engaged in and supportive of the development of the young people's new skills. For this reason, the intervention seeks to involve these actors, as well as mentors from the community who provide positive role models. In fact, evidence demonstrates that a strong attachment with a caring adult may help build resilience by building 'competence, confidence, character, connection and caring' (Lerner et al. 2005; p. 13). In line with the original *Engage in Education programme* goals, the intervention aims to develop the students' awareness of a range of adaptive communication skills and emotions and support their skills in interacting positively with others, in order to facilitate their engagement in more prosocial behaviours and less antisocial behaviour.

Programme delivery The intervention is delivered in three main components: group work sessions, one-to-one meetings and family support.

Group work consists of a set of 12 semi-structured one-hour long sessions facilitated by a trained 'core worker'.[1] The sessions are delivered utilising participative techniques (e.g., pair and group work activities and whole group discussions) aimed at encouraging the students' active involvement. The young people also agree to follow rules set by the group during discussions. Each session is structured around specific goals, which are outlined at the beginning of each session. Session content and the resources required for delivering each session (e.g., scheme of work, session plans, session worksheets) are described in a guidebook available to each core worker at the time of the training. Table 1 displays the curriculum and main goals of each of the 12-sessions.

Table 1 Catch 22 intervention sessions

Sessions	Main contents
**1. The skills I start with**	To learn effective communication skills. Participants are invited to think about their strengths and difficulties in regard to their communication strategies with teachers and peers.
**2. Managing difficult emotions**	To learn effective anger management skills. Participants are made aware of a range of emotions, the triggers for some emotions and some alternatives for managing them.
**3. Understanding conflicts**	To learn strategies for self-calming and de-escalating confrontations.
**4. I have choices**	To learn to appreciate the availability of different alternatives in a range of situations, to appreciate choices; their causes and effects.
**5. Check it out**	To learn to identify difficulties in comprehension; being aware of confusion by instructions; positive skills and attitudes to ask for extra explanations (e.g., interrupting appropriately).
**6. Different talk for different people**	To learn to adjust the way of talking depending on one's conversation partner and location. Develop an understanding of the difference between formal and informal communication exchanges.
**7. Looking back looking forward**	Evaluate personal performance and setting goals for the second part of the course.
**8. Co-operating with others**	To learn assertive communication skills in-group situations. Discussing with others in small groups, accepting others' opinion, changing personal opinions.
**9. Aggressive, Assertive, Passive**	To learn to understand and be aware of different styles of communication (aggressive, assertive, passive) and develop skills for adaptive, assertive interchange.
**10. Communication without talk**	To learn to understand body language and non-verbal signals. To be aware of potential biases based on non-verbal signs/stereotypes (dress, ethnicity, posture, etc.).
**11. I can change my world**	To learn to identify and acknowledge personal difficulties with classroom behaviour and identify strategies to improve.
**12. Summing up**	Final session summarizing the learning process, relevance of communication skills, personal achievements and personal challenges.

One-to-one work is designed to offer personalised support to each youth as well as reinforce concepts and skills learned during group sessions. The one-to-one meetings take place on a weekly basis during the school day, timetabled around the group sessions. The young person is given a list of skills that they rank, using this as a guideline they decide on up to three areas to work on. Throughout this process the core worker can help to prompt them to reflect on areas to focus on and also help them with how to structure this into written goals. The core worker will also structure their one-to-one activities/discussions around these target areas. These goals are reviewed in one-to-one sessions at various points throughout the intervention, new goals can be set if previous ones have been met. Example target areas include: calming down in arguments, listening to teachers, using positive body language. Thus, in the one-to-one meetings, led by core workers, the curriculum covered in the group sessions is adapted to each young persons' specific needs. This individual level approach ensures that there is a degree of autonomy for each core worker to tailor their delivery for each participant and source the appropriate support, whether academic, pastoral or familial.

Finally, when appropriate and necessary **family support** is offered. Core workers make home-visits, offering support in transferring families to suitable community services. The intervention intends to engage families in the task of supporting children to remain (or re-engage) in school and to improve their behaviours.

Core worker training The recruitment of core workers is an essential aspect of the intervention as its success is largely dependant on their relationship building abilities and delivery of the intervention. In August 2013, 11 core workers were recruited based on several criteria, in particular their previous experience of working with young people and schools, ability to understand the challenges of engaging positively with young people who have complex needs, as well as experience of assessing and formulating support plans for young people's achievement of learning outcomes. In September 2013, all core workers attended a four-week-long training and induction programme run by Catch22. This intervention training programme familiarised core workers with the organisation and its policies and procedures and equipped them with the relevant knowledge and practical skills to effectively deliver the 12-week intervention programme as well as to manage a caseload of young people though the one-to-one individual work.

During the staff training core workers were introduced to the delivery model and resources through presentations and workshops delivered by the service manager and I CAN Communication Advisors. Communication difficulties in young people with behaviour difficulties often go unrecognised (Gilmour et al. 2004; Ripley and Yuill 2005) and so an understanding of communication difficulties, how to identify and support them is crucial. Core workers were given a guidebook and resource pack that included the 12-week delivery model plan and scheme of work, a chart of the programme staffing structure and an example of the group and 1:1 session 'planning & evaluation' template. During the training month core workers take part in a variety of training activities, including an introduction to communication awareness training, behaviour management training, as well as instruction and practical experiences in using the intervention resources. For example, core workers are asked, in pairs, to review the group session delivery resources before deciding upon and planning a selected activity to run with the rest of the group. The core workers then role-play and run their activity with the other participating trainees. The group is then encouraged to provide feedback to each pair relating to their delivery style, use of resources or any other relevant observations.

I CAN 'Behaviour Talks' Workforce Development programme for schools

An additional component of the intervention is delivered by a partnering agency called I CAN. I CAN delivers a programme called "Behaviour Talks" to participating schools. Intervention schools are offered the I CAN Behaviour Talks workforce development programme. Behaviour Talks is a clear step-by-step programme of 'Communication Focused' activities and resources. It gives school staff the tools and confidence to identify and support the communication needs of young people with behaviour difficulties.

Control Group 'Light Intervention'

Schools in the control group are offered a one-off workshop delivered by trained corporate volunteers. These workshop sessions address employability skills of young people, provide insight into the world of work and facilitate discussions concerning employment.

Design

The trial is conducted and it will be reported in accordance with the requirements of the Consolidated Standards of Reporting Trials (CONSORT) Statement (Campbell et al. 2012).

Type of trial

The study design is a cluster-randomised controlled trial with randomisation at the school level. The sample consists of 36 schools, which are randomly allocated into one of two intervention conditions. Originally, we planned to complete baseline data collection in the month of September and randomise all schools at the end of September. Based on this plan all schools were going to be engaged with the intervention ('Intensive' or 'Light') for the duration of the entire academic year 2013/2014. The intervention was going to be delivered to Year 9 (50% of the schools) or Year 10 (50% of schools) students in each of the schools in Autumn 2013 and to the complementary Year group in each school in Winter/Spring 2014.

However, due to scheduling difficulties on the part of the schools, by the end of September we were only able to collect baseline information from 20 schools. As a result and following consultation with Catch22, EEF and GLA, the treatment delivery plan was revised and the schools were divided into two groups (Phases). Phase I schools were randomised and received the intervention in both Year groups in Autumn 2013 and Phase II schools were randomised later and received the intervention in both Year groups in Winter/Spring 2014. Please see Additional file 1: Table S1 for the data collection and intervention timeline for Phase I and II.

Twenty schools with available baseline data were randomised as planned at the end of September (constituting Phase I). The remaining 16 schools (constituting Phase II) were randomised on 15th of November. Please see Figure 1 for the CONSORT flowchart (Campbell et al. 2012; Moher et al. 2010) reporting school recruitment and randomisation.

The Phase I randomisation yielded 11 schools in the 'Intensive' intervention condition and 9 schools in the 'Light' intervention condition. Due to capacity limitations, the intervention provider was unable to deliver the intervention

to 11 schools (22 intervention groups) so one school was approached and accepted the proposal to receive the 'Intensive' intervention in Winter/Spring 2014. Thus, although 20 and 16 schools, respectively were randomised for Phase I and Phase II; 19 and 18 schools received the intervention corresponding to the Phase relevant intervention timeline.

Randomisation method

Randomisation was carried out through the process of 'minimisation' (Tavers 1974; Pocock and Simon 1975; Freedman and White 1976). This process was selected as it offers several advantages over pure random allocation, in particular that small-sample variation can lead to very imbalanced trials, some have even argued it is the 'platinum standard' for randomisation (Treasure and McRae 1998). The essence of the minimisation approach is that it does not rely solely on chance – it aims to reduce (i.e., minimise) differences in determinants of the outcome so that any remaining differences can be attributed to the outcome (Treasure and McRae 1998). To overcome the issue that pure minimisation is deterministic, the algorithms used also include a random component that reduces the chance of prediction – rather than favouring a reduction in imbalance scores, preference is given to allocation to treatment (Saghaei and Saghaei 2011). Thus, the minimisation algorithm is a flexible allocation method in which the allocation of each subject (e.g., individual or school) is influenced by the existing overall balance of allocated subjects (Saghaei and Saghaei 2011). One consequence of focusing on balance is that minimisation can lead to unequal sample sizes in treatment allocation arms.

Minimisation takes a series of steps (Saghaei and Saghaei 2011): (1) The first subject is allocated 'truly randomly'. (2) All following subjects are allocated hypothetically to both treatment and control groups and imbalance scores are calculated for each alternative. The question asked is: to which group would allocation of the next school make the two groups more balanced? (Altman and Bland 2005). If it makes no difference (i.e., the scores are tied) then allocation is again truly random. (3) Balance scores are compared for the alternative scenarios and the subject is allocated to the group that results in the 'least worst' imbalance score, but with 'treatment' being the preferred allocation. (4) Subsequent allocations use existing information to then repeat steps (2) and (3) until all subjects have been allocated. These steps do not guarantee perfect balance, but they do reduce the likelihood of imbalance versus simple randomisation. There are several software implementations of minimisation (Altman and Bland 2005). Here we use the open-source MinimPy software developed by Saghaei and Saghaei (2011).

In a simple random allocation model we expect to see that factors empirically or theoretically related to the outcome are 'balanced' between the arms of a trial. This might be assessed, for example, by exploring whether the proportion of males is roughly the same in each school. This is not the same as assessing whether such differences are statistically significant. There are several scores provided for assessing imbalance within MinimPy. We use the mean marginal balance score, with lower scores achieving greater balance. This can also be assessed by statistically testing differences between schools once allocation has been completed.[m]

Balancing schools

There are many variables that might be 'important' for school exclusion. We balanced on those variables originally used to select schools and based on prior research on factors strongly associated with the likelihood of exclusion: free school meal eligibility (FSM) and special educational need (SEN) derived from the 2012 school census data by the Department for Education (2012). In addition, as schools varied with respect to size and/or tailoring to only one gender, we also considered these two factors in the balancing of schools. Finally, we incorporated data from the baseline teacher questionnaire relating to assessed pupil behaviour (discussed below). Following the example in Altman and Bland (2005) we set out how each measure used in the minimisation process was created, presenting summary statistics for each measure in Table 2.

Proportion of children on free school meals

To be considered for the study, schools had to have > =28% of children currently eligible for free school meals (FSM) based on Department for Education (2012) data from the 2012 school census (Sutherland and Eisner 2014) meaning that the schools' intake is *a priori*, made up of children from poor backgrounds. The values of FSM for those schools eventually included in the trial ranged between 28-61%, with a median of 37%. We created a variable that took two values, splitting at 37%. Panel A of Table 2 shows the average proportion of FSM eligible children for each group.

Special Education Needs

The proportion of children classified as having special educational needs (SEN) ranged from 4.5-42.6%, with a median value of 12.05%. Schools with less than 12.05% were classified as 'low SEN' and those equal to or greater than 12.05% as having 'high SEN'. Panel B of Table 2 shows the distribution of these variables between the schools and the mean proportion with special education needs in both groups.

School gender

Department for Education data from the 2012 school census stated that we had a mixture of eight single sex

Table 2 School variables used for minimisation

Panel A. FSM group	Mean % FSM eligible	Freq.
0 < 37%	32	17
1 > =37%	45	19
Total	38.9	36
**Panel B. SEN group**	**Mean % SEN**	**Freq.**
0 < 12.05%	8.0	18
1 > =12.05%	19.3	18
Total	13.7	36
**Panel C. School gender**	**% of schools**	**Freq.**
Mixed sex	72	26
Single sex	28	10
Total	100	36
**Panel D. Year 9/10 Cohort sizes**	**Mean *n* pupils**	**Freq.**
Small <250	747	8
Medium 250-400	900	13
Large >400	1246	15
Total	1010	36
**Panel E. Teacher questionnaire**	**Mean PCA score**	**Freq.**
0 < Mean ASB	-.760	17
1 > Mean ASB	.741	19
Total	-.045	36

schools (three all-boys, five all-girls) and thirty mixed sex schools. However, upon closer inspection, via examining the proportion of male pupils in each school, some schools listed as 'mixed sex' in Department for Education data were in fact all male. The median value for percent male was 56%, but the range for supposed mixed sex schools, was between 45-101% with 101% being a school that was above capacity and with an all-male intake. 'Mixed sex' schools that consisted of all-male pupils were classified as 'single sex' for the purposes of randomisation.[n] This resulted in ten schools classified as 'single sex'. Panel C of Table 2 reports the number of schools classified as 'mixed' or 'single sex'.

School size (total number of pupils enrolled)

Schools were split into three groups (small, medium and large) based upon information returned by schools on the current size of their Year 9 and 10 cohorts. This partly determined how many pupils the research team requested were put forward for the intervention. Schools were classified into 'small' (less than 250 pupils), 'medium' (250–400) or 'large' (more than 400) based on the number of pupil enrolled in each year groups. Of the 36 schools 8 (22%) are small, 13 (36%) are medium and 15 (42%) are large. Panel D of Table 2 displays the average number of pupils in the school for each of these groups.

Teacher questionnaire data

To ensure balance on factors directly relating to exclusion, we also incorporated data from the baseline teacher questionnaire data. Section 1 of the questionnaire (see Table 3) consisted of 15 questions relating to pupil behaviour, both positive and negative. In order to incorporate this information, we use principle component analysis to reduce the dimensionality of the data. This resulted in questions clustering around two dimensions, what we termed 'Anti-Social Behaviour' (ASB) and 'Pro-Social Behaviour' (PSB). This information was then aggregated to the school level and split at the mean. Only the 'Anti-Social Behaviour' score was used in minimisation. Panel E of Table 2 shows the number of schools designated as 'high' and 'low' ASB.

Sample size calculations

In an experiment (e.g., RCT) we are asking whether two groups are the same (Null Hypothesis – H_0) or different (Alternative Hypothesis – H_A). "Power" is the probability of detecting a difference (e.g., due to the effect of a treatment) between groups, if it exists. Therefore, when designing an experiment, our goal is to make sure that we have a large enough sample size to ensure a high probability to be able to detect differences. This typically means having a large enough sample, however; other factors also influence power: s*ample size, effect size, significance level,* and the *statistical test used* (Cohen 1988; Hedges and Rhoads, 2010) set out some additional factors that influence power in complex designs such as cluster-randomised trials. In brief, these are: (i) *the number of clusters* – when there is statistical dependence among scores within a cluster (e.g., pupils in classrooms or schools), power is no longer purely a function of how many individuals there are in a trial, but is much more strongly affected by the number of clusters, which is always a smaller number. This reduced sample size in turn affects statistical power (as above). (ii) *Intra-class correlation* (ICC). The ICC is the proportion of the variance of the dependent variable that occurs *between* clusters. If the differences in the data are not due to the differences between the clusters, then the ICC will be 0 and the effective sample size for the study will be all the individuals who participated in the study. If, however, all of the differences are due to differences between clusters then the ICC will be 1 and the effective sample size will be the number of clusters. In reality, the ICC will be somewhere between 0 and 1, therefore, the effective sample will be somewhere between the number of individuals and clusters. (iii) *Baseline adjustment*. Assessment of the dependent variables (i.e., outcome variables) at baseline as well as following treatment allows controlling for the baseline levels of each outcome and thus increases the operative sample size (the essence of this is that it reduces the 'noise' between the groups and thus makes it

Table 3 Teacher questionnaire - anti-social behaviour items

For the following questions, please indicate how often in the past YEAR this young person has…	Never	Rarely (1 to 2 times)	Sometimes (3 to 10 times)	About once a month	About once a week	Almost every day
… Physically attacked another young person.	□	□	□	□	□	□
… Verbally abused/ threatened another young person.	□	□	□	□	□	□
… Physically attacked an adult/s.	□	□	□	□	□	□
… Verbally abused/ threatened an adult/s.	□	□	□	□	□	□
… Abused others because of their race.	□	□	□	□	□	□
… Deliberately disrupted teaching.	□	□	□	□	□	□
… Engaged in sexually inappropriate behaviours.	□	□	□	□	□	□
… Used drugs and/or alcohol.	□	□	□	□	□	□
… Damaged the school's or somebody else's property at school.	□	□	□	□	□	□
… Was rude and belligerent toward me.	□	□	□	□	□	□
… Stole something at school.	□	□	□	□	□	□

easier to detect differences). In order to maximize power based on baseline adjustment we need to utilize measures with acceptable test-retest reliability.

Minimum detectable effect size

Logistical restrictions on the maximum number of schools and pupils within schools that could receive the intensive intervention mean that instead of calculating an optimum sample size to detect a desired effect size, we are instead calculated the minimum detectable effect size (MDES). Bloom (1995; cited in Spybrook et al. 2011; p. 7) 'defines the MDES as the smallest true effect that can be detected for a specified level of power and significance level for any given sample size'. At the outset of study design, we used the Optimal Design software (Raudenbush et al. 2011) with 40 schools (J); 20 pupils (n) within each school; a desired power of .80; an alpha of .05; an assumed ICC of 10%; assuming no correlation between baseline and post-intervention data; and no level two measures.[o] Using these parameters the (conservative) estimate of MDES is d = .35, which is in the 'small' to 'medium' range (Cohen 1988) see Figure 2. With the addition of baseline and school level covariates the MDES will reduce accordingly as power increases.[p]

Blinding

Screening data and baseline teacher reports were collected in July 2013 prior to the end of the 2012/2013 academic year to ensure that the teachers had sufficient exposure to and experience with the pupils to reliably report on their behaviour. Some schools (n = 6) returned data after the new school year had started but before randomisation. One school failed to submit any teacher questionnaires, hence was randomly allocated a score for minimisation. As both the research team and teachers were blind to whether their school is in the treatment or control condition we achieved a double blind design for baseline data collection.

Ethics statement

Ethics/code of conduct

The project and the consent procedure described below were approved by the Institute of Criminology Ethics Review Committee on 20 May 2013 (approval letter available upon request). All data for the project will be held in compliance with the 1998 Data Protection Act. All schools involved in the study signed data sharing agreements with the University (example data sharing agreement available upon request).

Teacher consent

Teachers were asked to complete an informed consent form when filling out the online and paper versions of the study questionnaire.

Parental consent

Following identification of the (average of) 20 young people per school, consent was sought from parents. After much deliberation with colleagues within the University, as well as consultation with teachers, local council education officers, the intervention provider (Catch22) and the Educational Endowment Foundation, we decided that a parental 'opt-out' approach would best fit the study design, the target group of (high risk) young people and is in keeping with how schools routinely approach the provision of additional support. These letters were prepared by the research team but amended/sent by the schools themselves and signed by the Headteacher or other school representative. Parents

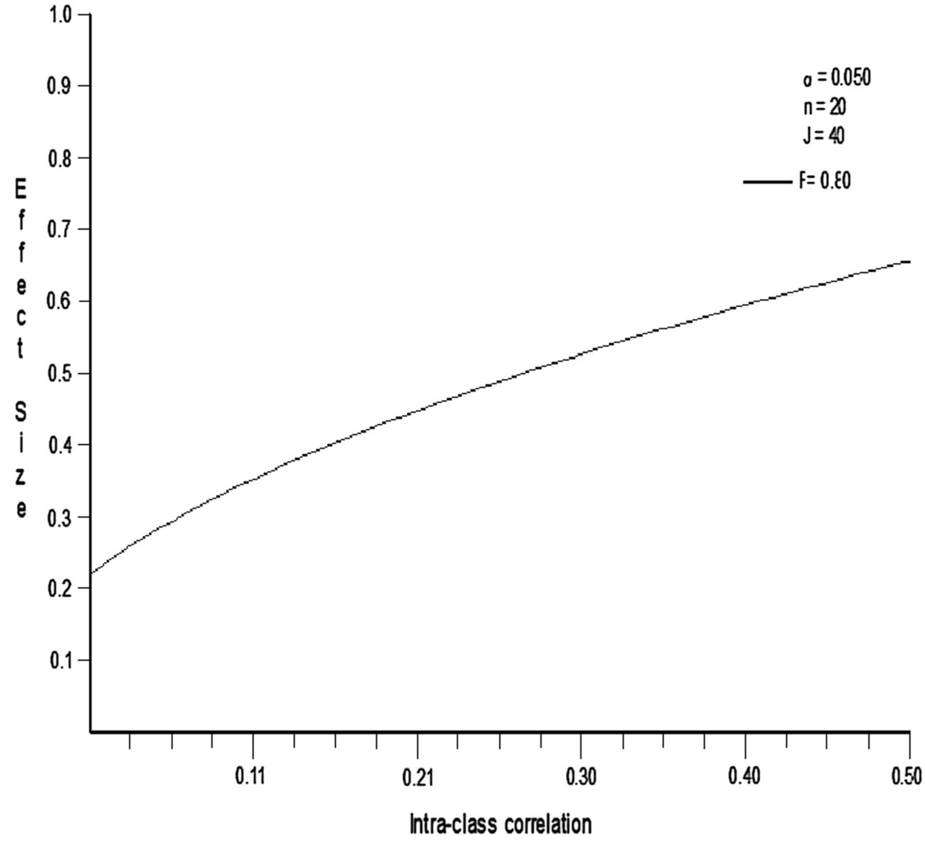

Figure 2 Minimum detectable effect size (MDES) for LEIP.

were given one week to advise the research team by contacting the school (either by post or phone) and indicating that they wish to opt out of the study. Of the approximately 800 letters sent to parents, 15 parents/guardians indicated that they wished to opt their child out of the study.

Young person assent

Prior to completing any questionnaires, participants were presented with an Information Sheet/Assent Form. Fieldworkers read out the study information portion of the assent form to the group and make sure that each young person understood what was being asked of them. As we plan to follow up participants beyond the life of the study and link their data to government records, they were asked to tick separate boxes to consent to linking self-reported data to official records from the Ministry of Justice and Department for Work and Pensions. We reiterated that we would not share any of this information with the school, their parents, the police or anyone else, that their responses to the questionnaires are confidential, and that all of their information will be anonymised. Once this was completed and the young people had the opportunity (or been prompted) to ask questions, they were asked to confirm their willingness to participate by signing the forms. When parental consent and young person assent were opposed then parental consent was considered.

Fieldwork data collection procedures

Staffing For the *baseline data collection,* 15 fieldworkers (FW) were recruited from University College London (UCL; n = 14) and the London School of Economics (LSE; n = 1). Following a thorough selection procedure all FWs attended a two-day training course on administration of the questionnaires as well as conduct with the young people, led by the research team.

For the *post-intervention data collection* three of the original FWs who were still available were invited back to participate in the collection of the post-intervention data. As the post-intervention data collection overlapped with the exam period at UCL, we widened the recruitment net to include other Universities (Departments of Psychology and other social science fields) throughout London. Fifteen new FWs were recruited from Royal Holloway University (n = 2), University of Roehampton (n = 4), Kingston University (n = 5), Goldsmiths University (n = 3) and one visiting student from Deakin University in Australia. Based on feedback from FWs following the baseline data collection the training and all training material was delivered in a

one-day training session. All FWs were provided with a handbook containing details of the study and procedures to follow in the field. Regular contact is maintained with FW staff by the central research team to provide support as well as monitor and facilitate the data collection process.

Data Collection Timeline Baseline data was collected from teachers and the young people prior to randomisation of all schools as specified below. The post-intervention data collection from both teachers and young people is currently (March – May, 2014) ongoing for Phase I schools and is planned to be completed in June – July for Phase II schools. The post-intervention data collection is being completed in two stages to follow approximately one month after the completion of the intervention in the intervention groups in each Phase. Both teachers and young people are asked to report about the young people's behaviours and experiences from the previous month (see Additional file 1: Table S1 - Timeline).

Teacher questionnaires Teachers completed an online or paper and pencil version of an assessment with respect to potential participants. Baseline teacher reports were collected in June/July 2013 for the majority of schools (n = 30). For a few 'hard to reach' schools and those coming later into the study, teacher questionnaires were returned in September 2013. Schools were asked that the form tutors or the teacher who knew the children best should complete the questionnaire. This was to increase the probability that the teachers being asked have sufficient knowledge of the young people participating in the study. In all schools, this assessment was collected prior to randomisation. The teacher questionnaire asks about the young person's behaviour problems, prosocial behaviours and likely reactions to punishment disciplinary actions that had been taken against that child, as well as the quality of the teacher-student relationship. Any teacher baseline data relating to young people who will not be included in the study (i.e., any reserve young people) will not be kept beyond the lifetime of the study. Form tutors (note that these may be different teachers than at baseline) will be approached to provide the same information about the young people.

Young people Baseline measurements were carried out in groups of 10–20 young people, overseen by two to four trained FWs, with a maximum of five young people per FW. Both at baseline and at post-intervention data collection, the young people complete a questionnaires as well as a computerized educational abilities measure. FWs were instructed to first explain the study to the young people and ensure that they understand it, administer the young person assent form (at baseline) and then proceed with the administration of the paper and pencil and online questionnaires. Whenever possible both assessments are carried out on the same day and young people not present during the initial group-based baseline data collection are followed up by pairs of FWs individually.

Young person questionnaire The young people complete a paper and pencil questionnaire, consisting of 144 questions rated (mainly) on Likert Scales tapping the young people's behaviours, emotions, relationships with peers and teachers, as well as communication skills. The duration of the administration of the questionnaire is 30–40 minutes.

The questionnaire and procedure were first piloted in a local comprehensive school on a sample of 19 young people. It was then adjusted to account for the difficulties to maintain the attention of groups of young people. Scripted instructions, which were read out by the FWs to the group of young people, were also added.

Educational abilities measure The young people also complete a computer-administered measure developed by the Centre for Evaluation and Monitoring at Durham University.[q] This measure provides a standardised, adaptive, curriculum free assessment of the young people's maths and verbal abilities. The duration of administration is up to one hour.

Outcome measures

Outcomes are designed to reflect the main domains targeted by the intervention. A multi-informant approach is adopted whereby data will be collected from official records, teachers and the study participants. We anticipate that the planned intervention will have positive effect on **five** interrelated primary outcomes:

1. Improve communication skills as the main proximal mechanism targeted by the intervention.
2. Reduce behavioural problems, including the likelihood of school exclusions up to the end of compulsory schooling.
3. Improve academic outcomes, in particular the number of GCSEs being sat and GCSE exam results.
4. Reduce the risk of becoming NEET in the years after compulsory schooling.
5. Reduce the risk of arrests and criminal convictions during and after compulsory schooling.

In addition, given the emphasis placed by the intervention programme on communication skills, we expect these to improve regardless of other outcomes. Table 4 gives a list of the outcomes and measures. We plan to follow up participants for at least two years after the intervention via administrative records in various government agencies (e.g., Ministry of Justice; Department for Education; Department for Work and Pensions), but this falls outside of the remit of this trial.

Table 4 Outcomes and source

	Source
**A) Academic outcomes**	
Academic Attainment Test, Verbal and Maths	Online academic tests administered in schools
Academic Achievements	School records
Attendance	School records & teacher assessments
**B) Interpersonal skills**	
Student communication skills	Self-Report
Student communication skills	Teacher assessment
Student prosocial skills	Self-Report
Student prosocial skills	Teacher assessment
Student-teacher relationship	Self-report
Student-teacher relationship	Self-report
**C) Behaviour problems**	
School exclusions	School records
Bullying perpetration	Self-report
Delinquency	Self-report
Antisocial behaviour	Self-report
Antisocial behaviour	Teacher assessment

Analysis plan
To reduce or minimise threats to internal validity, selection bias and post-randomisation biases (Shadish et al. 2002) the trial was designed, is conducted and will be reported according to CONSORT standards (Campbell et al. 2012). These consist of a 'quality assurance' checklist for such studies.

Baseline equivalence
In a first step *t*-tests (or equivalent for proportions) will be calculated to examine differences in all baseline measures, socio-demographic measures and mediators between the control and the treatment group. Test of equivalence at baseline will take into consideration the clustering of the data. The hypothesis is that if the randomisation was successful the outcomes will not differ at baseline.

Attrition and missing values
A CONSORT diagram will document the loss of participants between the baseline and post-intervention assessments. We expect to keep attrition to approximately 15% of the baselined sample. Ideally, we would have oversampled by 15% to increase the sample size required to account for attrition. However, this proved difficult for two reasons: a) schools reported not having any more young people who would meet criteria and b) the intervention provider was only able to accommodate a maximum of 12 young people per intervention group and a maximum of 20 intervention groups per phase.

Clustering
To take into account the clustering of the subjects within schools we will use models that assume correlated errors within each cluster such as multilevel models, or models with cluster-robust standard errors.

Missing values
Missing values due to attrition or non-response will be imputed. The imputation strategy will depend on the extent of missingness (e.g., what proportion of our data matrix is missing) and the missingness mechanism.

Outcome assessment
Initial analyses of all outcomes will be conducted on an intention-to-treat (ITT) basis, i.e., all participants allocated to the treatment and control conditions will be included. We are planning a covariate-controlled assessment of differences in each outcome at baseline. Analyses will be conducted on all outcomes listed in Table 4. We will conduct two-tailed hypothesis tests against the standard 5% alpha level, applying family-wise error correction where necessary (e.g., Bonferroni). As noted above, clustering will be taken into account via multilevel models or by using cluster-robust standard errors.

Sub-group analyses
Subsequent to the main ITT analyses we are planning a number of subgroup analyses:

– Exposure to treatment: Data will be collected on whether and what proportion of the planned intervention was received by each young person. We expect a dose-response relationship in that children who more fully participated in the intervention show more change in the expected direction. To test this hypothesis we will carry out a subgroup analysis by levels of exposure to treatment.
– Implementation: Data will be collected on the implementation process. We will analyse whether higher implementation quality is associated with better treatment effects.
– Engagement with school: Baseline measures will be taken on the children's engagement with the school and the teacher. We hypothesize that children who are more engaged with the school and the teacher will show greater improvements than children who are less engaged.
– Initial behaviour: Research suggests that higher levels of initial problem behaviour are often associated with better effects. We will examine intervention effects by baseline level of behaviour and communication problems.
– Year group and sex: We will also examine whether the intervention had different effects by year group

and sex, although we do not have theory-led hypotheses about sex and age-specific differences in the effects of the intervention.

Furthermore, we plan analyses of mediating mechanisms. More specifically, we will examine whether improvements in communication and social skills mediate effects on more distal behaviour and academic outcomes.

Process evaluation methods

In recent years researchers and policy makers have argued that in addition to answering the question of "Does an intervention work?" in randomised treatment trials it essential to also carry out process evaluation to answer questions such as "What works?", "Why does it work/does not work?" (Oakley 2006) and "What works for whom?" (Pawson and Tilley 2004). Process evaluations focus on documenting and evaluating the implementation of the intervention, dosage of each component, adherence to treatment, treatment delivery, contextual factors that may influence the intervention and possible subgroup effects (Wight and Obasi 2002). Each of these components may influence as well as help in the interpretation of the outcome results (Durlak and DuPre 2008). According to Oakley (2006) process evaluations are particularly important in multi-site trials, in which the same intervention may be delivered in more or less different ways across the different sites, which may lead to systematic differences. Therefore, in the current evaluation, Catch22 will provide regular data on the programme delivery relating to: planned interventions, compliance with the intervention (e.g., attendance, dropout, disruption), any problems with maintaining fidelity, any deviations from what was planned with documented explanations and completion. We have also carried out semi-structured interviews with the Catch22 core workers providing the Intensive intervention to find out more about their experiences with their training and implementation of the EiE London programme. This information will be utilized to evaluate the above listed processes identified as key in process evaluation and will be also be used in relevant subgroup analyses.

Trial status

The trial is in the initial stages of post-intervention data collection.

Discussion

The UK has perhaps one of the highest rates of school-exclusions. Already in 1998 the (UK Social Exclusion Unit) concluded that the school exclusion levels have reached a 'crisis point'. They suggested that "The thousands of children not in school on most school days have become a significant cause of crime. Many of today's non-attenders are in danger of becoming tomorrow's criminals and unemployed" (Macrae et al. 2003; p. 91). Compared to other countries, for example in Switzerland where school-exclusions are utilized as a 'treatment' strategy, in the UK school-exclusions are utilized largely as one of the more extreme types of disciplinary measures (Parsons 2005). Despite recent evidence from other countries suggesting that school-exclusions are not only non-effective in achieving behavioural and/or educational improvements in young people, but may in fact be harmful (Gazeley 2010; Osler and Vincent 2003), they continue to be widely used in schools throughout the UK.

The current study builds on preliminary evidence on the effectiveness of the Catch22 intervention carried out by the Department for Education in 2011/13 (Catch22 2013b). It is a large-scale field trial, which aims to provide and solidify the evidence of the effectiveness (with respect to both behavioural and educational outcomes) of this intervention specifically targeting difficult to engage young people at the highest risk for school exclusion. It also aims to gather a wide range of information about this high-risk group of young people, which will allow for the better understanding of their experiences at and outside of school. Based on this information, the study also aims to elucidate some of the processes which may link school exclusions to later adverse behavioural and/or educational outcomes.

Furthermore, in addition to the assessment of short-term effects, long-term follow-up of these young people based on official records will enable the evaluation of the long-term effects of the intervention on their employment as well as possible engagement in antisocial/criminal behaviours.

The study has further strengths in that, to our knowledge, it is the first cluster-randomised controlled trial of a preventive intervention for a very specific group of young people at the highest risk for school exclusion in the UK. It is also an independent evaluation, in which the programme is implemented and evaluated by two separate teams funded by two different funding sources. Moreover, the study has high external validity as, amongst others, it models a recruitment process parallel to the one generally utilised by the Catch22 intervention. As a result, the findings from this study will be generalizable to a wide population of young people at high-risk for school exclusion to who may benefit from this intervention.

Endnotes

[a]These are also known as fixed term exclusion or 'suspension'; all three terms are used interchangeably throughout.

[b]Other legislation relevant to school exclusion is given here: https://www.gov.uk/government/publications/school-exclusion.

[c]Boys are around three times more likely to receive a permanent or fixed period exclusion than girls (similar to

the previous year). More starkly pupils eligible for free school meals are four times more likely to receive a permanent exclusion than those not eligible and the fixed period exclusion rate for these children is around three times higher than the rate for those not eligible (Department for Education 2012).

[d]Physically assaulting a pupil or an adult (20.5%), 'persistent disruptive behaviour' (24.1%), verbal abuse or threatening behaviour towards an adult or pupil (25.5%) (Department for Education).

[e]AKA 'school climate' in the US, see e.g.: http://www.schoolclimate.org/climate/.

[f]Specifically, being NEET (Not in Education, Employment or training).

[g]This cut-point of 28% was determined by the EEF on the basis of it representing above average levels of deprivation within London.

[h]http://educationendowmentfoundation.org.uk

[i]The EEF's aim is to narrow the gap in attainment for pupils from disadvantaged backgrounds.

[j]http://www.catch-22.org.uk/Engage-in-Education

[l]Core workers are full-time staff responsible for the management of a caseload of young people deemed "at risk of exclusion". Their role is to a) assess those young people and develop an agreed action plan of bespoke interventions that meets their needs, review this regularly and feedback to stakeholders as required; b) to work with colleagues in the delivery of small group activities and workshops for young people and the wider community throughout the lifecycle of the project; c) to conduct all necessary administration and evaluation duties and to work in line with Catch22 policies at all times.

[m]Ideally, we would include balancing variables as covariates in later analyses because these capture the selection process.

[n]The two schools were one secondary school and an 'all boys' school. It may seem obvious that an all-male school could only have male pupils, but it might be that the school has a mixed sex sixth form. Equally, the DfE classification could have been incorrect.

[o]Meaning that both the test-retest correlation and school level variance explained were assumed to be zero.

[p]With $J = 35$ and the same parameters as above, MDES is $d = .38$; with $J = 30$, MDES is $d = .40$.

[q]http://www.cem.org

Additional file

Additional file 1: Table S1. Project Timeline – intervention and data collection.

Competing interests

As this trial is an independent evaluation, the Intervention Team and the Evaluation Team consist of different groups of people funded by different external funding sources. Rosanna Hall is part of the Intervention Team (funded by the Education Endowment Foundation), in her role she oversees all aspects of the delivery of the intervention but had no role in the design of the evaluation. The remaining authors are all part of the Evaluation Team (funded by the European Commission and Greater London Authority).
As such these authors declare that they have no competing financial or non-financial interests.

Authors' contributions

IO participated in the design of the study, organization and supervision of data collection and training, preparation of this manuscript. AS helped secure funding for the project, participated in the design of the study, organization and supervision of data collection and training, preparation of this manuscript. SV participated in collecting information to describe the intervention section of this protocol/manuscript. SS participated in collecting information for key aspects of this manuscript and contributed to first drafts of some parts. LN participated in collecting information for key aspects of this manuscript and contributed to first drafts of some parts. RH participated in the development, implementation and description of the intervention. ME secured the funding for the project, participated in the design of the study as well implementation of the study; has been involved in revising this manuscript for important intellectual content. All authors read and approved the final manuscript.

Acknowledgements

We thank the European Commission, which via a Social Experimentation Grant (EC reference VS/2012/0345) provided funding to the Greater London Authority for this specific project in collaboration with Professor Manuel Eisner, University of Cambridge. The information contained in this publication does not necessarily reflect the position or opinion of the European Commission. We also thank the Education Endowment Foundation, which provided funding for the implementation of the Engage in Education – London programme, the target intervention of this trial.

Author details

[1]Institute of Criminology, University of Cambridge, Sidgwick Avenue, Cambridge CB3 9DA, UK. [2]Catch22, Unit 4, First Floor, 1-3 Stratford Office Village, 14/30 Romford Road, Stratford, London E15 4EA, UK.

Received: 3 July 2014 Accepted: 18 July 2014
Published: 15 August 2014

References

Altman, DG, & Bland, JM. (2005). Treatment allocation by minimisation. *British Medical Journal, 330*(7495), 843.
Boxford, S. (2006). *Schools and the Problem of Crime*. Willan: Cullompton, Devon.
Campbell, MK, Piaggio, G, Elbourne, DR, & Altman, DG. (2012). Consort 2010 statement: extension to cluster randomised trials. *British Medical Journal, 345*, e566.
Carr, EG, & Durand, VM. (1985). Reducing behaviour problems through functional communication training. *Journal of Applied Behavior Analysis, 18*(2), 111–126.
Catch22. (2013a). Transforming lives, transforming communities. [Retrieved from http://www.catch-22.org.uk/wp-content/uploads/2013/11/Impact-report-2012-2013.pdf]
Catch22. (2013b). Evaluation of Engage in Education, Department for Education. [Retrieved from http://www.catch-22.org.uk/Engage-in-Education]
Centre for Social Justice. (2011). No Excuses - a review of Educational Exclusion. [Retrieved from http://www.centreforsocialjustice.org.uk/UserStorage/pdf/Pdf%20reports/CSJ_Educational_Exclusion.pdf]
Clegg, J, Stackhouse, J, Finch, K, Murphy, C, & Nicholls, S. (2009). Language abilities of secondary age pupils at risk of school exclusion: A preliminary report. *Child Language Teaching and Therapy, 25*(1), 123–140.
Cohen, J. (1988). *Statistical power analysis for the social sciences (2nd edition)*. New Jersey: Lawrence Erlbaum Associates.
Cohen, LE, Felson, M, & Land, KC. (1980). Property crime rates in the United States: A macro dynamic analysis, 1947–1977; with ex ante forecasts for the mid-1980s. *The American Journal of Sociology, 86*, 90–118.
Department for Education. (2012). Permanent and fixed-period exclusions from schools in England: academic year 2010 to 2011. In London: Department for Education [http://www.education.gov.uk/rsgateway/DB/SFR/s001080/sfr17-2012.pdf]

Department for Education. (2013a). Permanent and fixed period exclusions from schools in England: 2011 to 2012 academic year. In London: Department for Education [Retrieved from https://www.gov.uk/government/publications/permanent-and-fixed-period-exclusions-from-schools-in-england-2011-to-2012-academic-year]

Department for Education. (2013b). School discipline and exclusions. In London: Department for Education [https://www.gov.uk/government/publications/permanent-and-fixed-period-exclusions-from-schools-in-england-2011-to-2012-academic-year]

Department for Education. (2013b). Schools, uniform. In London: Department for Education [Retrieved from http://www.education.gov.uk/popularquestions/schools/Uniform/a005643/school-uniform]

Dupper, DR, Theriot, MT, & Craun, SW. (2009). Reducing Out-of-School Suspensions: Practice Guidelines for School Social Workers. *Children and Schools, 31*, 6–14.

Durlak, JA, & DuPre, EP. (2008). Implementation matters: a review of research on the influence of implementation on program outcomes and the factors affecting implementation. *American Journal of Community Psychology, 41*(3–4), 327–350.

Ellis, P. (2013). Final Evaluation of Engage in Education – A Department for Education funded pilot programme delivered by Catch22 and partners (2011–2013). [http://www.catch-22.org.uk/wp-content/uploads/2013/11/Engage-in-Education-Final-evaluation-Executive-summary-June-2013.pdf]

Freedman, LS, & White, SJ. (1976). On the use of Pocock and Simon's method for balancing treatment numbers over prognostic factors in the controlled clinical trial. *Biometrics, 32*, 691–694.

Galloway, D, Martin, R, & Wilcox, B. (1985). Persistent absence from school and exclusion from school: the predictive power of school and community variables. *British Educational Research Journal, 11*(1), 51–61.

Gazeley, L. (2010). The role of school exclusion processes in the re-production of social and educational disadvantage. *British Journal of Educational Studies, 58*(3), 293–309.

Gilbertson, D. (1998). Exclusion and crime. In N Donovan (Ed.), *Second Chances: Exclusion from school and equality of opportunities*. London: New Policy Institute.

Gilmour, J, Hill, B, Place, M, & Skuse, DH. (2004). Social Communication Deficits in Conduct Disorder: a clinical and community survey. *Journal of Child Psychology & Psychiatry, 45*(5), 967–978.

Graham, J. (1988). Schools, disruptive behavior and delinquency - a review of research. In *Research Study 96*. London: Home Office.

Hayden, C. (2009). Deviance and violence in schools: A review of the evidence in England. *International Journal of Violence and School, 9*, 8–35.

Hedges, L, & Rhoads, C. (2010). *Statistical Power Analysis in Education Research*. Washington: National Center for Education Research, Institute of Education Sciences, US Department of Education.

Jaffee, SR, Strait LB, O, & Candice, L. (2012). From correlates to causes: Can quasi-experimental studies and statistical innovations bring us closer to identifying the causes of antisocial behavior? *Psychological Bulletin, 138*(2), 272–295.

Lerner, RM, Almerigi, JB, Theokas, C, & Lerner, JV. (2005). Positive youth development a view of the issues. *The Journal of Early Adolescence, 25*, 10–16.

Macrae, S, Maguire, MEG, & Milbourne, L. (2003). Social exclusion: Exclusion from school. *International Journal of Inclusive Education, 7*(2), 89–101.

Massey, A. (2011). *Best behaviour: School discipline, intervention and exclusion.* [Retrieved from http://www.policyexchange.org.uk/images/publications/pdfs/Best_Behaviour_Apr_11.pdf]

McAra, L, & McVie, S. (2010). Youth crime and justice: Key messages from the Edinburgh study of youth transitions and crime. *Criminology and Criminal Justice, 10*(2), 179–209.

Meltzer, H, Gatward, R, Corbin, T, Goodman, R, & Ford, T. (2003). *Persistence, onset, risk factors and outcomes of childhood mental disorders*. London: Office of National Statistics.

Moher, D, Hopewell, S, Schulz, KF, Montori, V, Gøtzsche, PC, Devereaux, PJ, Elbourne, D, Egger, M, & Altman, DG. (2010). CONSORT 2010 Explanation and Elaboration: updated guidelines for reporting parallel group randomised trial. *British Medical Journal, 340*, c869.

Munn, P, & Lloyd, G. (2005). Exclusion and excluded pupils. *British Educational Research Journal, 31*(2), 205–221.

Munn, P, Lloyd, G, & Cullen, MA. (2000). *Alternatives to Exclusion from School*. London: Paul Chapman Publishing Ltd.

Oakley, A. (2006). Process evaluation in randomised controlled trials of complex interventions. *British Medical Journal, 332*, 413–416.

Osler, A, & Vincent, K. (2003). *Girls and exclusion*. London, Routhledge Falmer: Rethinking the agenda.

Parsons, C. (2005). School exclusion: The will to punish. *British Journal of Educational Studies, 53*(2), 187–211.

Pawson, R, & Tilley, N. (2004). Realistic Evaluation. In S Matthieson (Ed.), *Encyclopedia of Evaluation*. Newbury Park: Sage.

Pocock, SJ, & Simon, R. (1975). Sequential treatment assignment with balancing for prognostic factors in the controlled clinical trial. *Biometrics, 31*, 103–115.

Raudenbush, SW, Spybrook, J, Congdon, R, Liu, X, & Martinez, A. (2011). Optimal design software for multi-level and longitudinal research (Version 3.01). [Software]. [http://www.wtgrantfoundation.org]

Ripley, K, & Yuill, N. (2005). Patterns of Language Impairment and Behaviour in Boys Excluded from School. *British Journal of Educational Psychology, 75*(1), 37–50.

Rosenbaum, PR, & Rubin, DB. (1983). The central role of the propensity score in observational studies for causal effects. *Oxford Journals, 70*(1), 41–55.

Rosenbaum, PR, & Rubin, DB. (1985). Discussion of 'On State Education Statistics': A difficulty with regression analyses of regional test score averages. *Journal of Educational Statistics, 10*(4), 326–333.

Rosenthal, R, & Jacobsen, L. (1968). *Pygmalion in the Classroom: Teacher expectation and pupils' intellectual development*. New York: Holt, Rinehart & Winston.

Rutter, M, Maughan, B, Mortimore, P, & Outston, J. (1979). *Fifteen Thousand Hours: Secondary Schools and their Effects on Children*. Cambridge, M.A.: Harvard University Press.

Saghaei, M, & Saghaei, S. (2011). Implementation of an open-source customizable minimization program for allocation of patients to parallel groups in clinical trials. *Journal of Biomedical Science and Engineering, 04*(11), 734–739.

Shadish, WR, Cook, TD, & Campbell, DT. (2002). *Experimental and Quasi-Experimental Designs for Generalized Causal Inference (2nd edition)*. Boston: Houghton-Mifflin.

Sherman, LW. (1993). Defiance, Deterrence and Irrelevance: A Theory of the Criminal Sanction. *Journal of Research in Crime and Delinquency, 30*, 445–473.

Sparkes, J. (1999). Schools, education and social exclusion. CASEpaper, 29. [Retrieved from http://eprints.lse.ac.uk/id/eprint/6482]

Speilhofer, T, Benton, T, Evans, K, Featherstone, G, Golden, S, Nelson, J, & Smith, P. (2009). Increasing participation: Understanding young people who do not participate in education or training at 16 or 17. [Retrieved from https://www.nfer.ac.uk/publications/PEJ01/PEJ01_home.cfm]

Spybrook, J, Raudenbush, SW, Congdon, R, & Martınez, A. (2011). Optimal design for longitudinal and multilevel research: Documentation for the "Optimal Design" software. [http://www.wtgrantfoundation.org]

Sutherland, A, & Eisner, M. (2014). The Treatment Effect of School Exclusion on Unemployment. SSRN. http://dx.doi.org/10.2139/ssrn.2380956.

Tavers, DR. (1974). Minimization: A new method of assigning patients to treatment and control groups. *Clinical Pharmacological Therapy, 15*, 443–453.

Treasure, T, & McRae, KD. (1998). Minimisation: the platinum standard for trials? Randomisation doesn't guarantee similarity of groups; minimisation does. *British Medical Journal, 317*(7155), 362–363.

Van Daal, J, Verhoeven, L, & Van Balkom, H. (2007). Behaviour problems in children with language impairment. *Journal of Child Psychology and Psychiatry, 48*(11), 1139–1147.

Wight, D, & Obasi, A. (2002). Unpacking the "black box": the importance of process data to explain outcomes. In J Stephenson, J Imrie, & C Bonell (Eds.), *Effective sexual health interventions: issues in experimental evaluation* (pp. 151–166). Oxford: Oxford University Press.

(1998). UK Social Exclusion Unit. [Retrieved from http://webarchive.nationalarchives.gov.uk/+/http:/www.cabinetoffice.gov.uk/media/cabinetoffice/social_exclusion_task_force/assets/publications_1997_to_2006/seu_leaflet.pdf]

doi:10.1186/s40359-014-0024-5
Cite this article as: Obsuth *et al.*: **London Education and Inclusion Project (LEIP): A cluster-randomised controlled trial protocol of an intervention to reduce antisocial behaviour and improve educational/occupational attainment for pupils at risk of school exclusion.** *BMC Psychology* 2014 **2**:24.
