# Supplementary material for: London Education and Inclusion Project (LEIP): Exploring Negative and Null Effects of a Cluster-Randomised School-Intervention to Reduce School Exclusion—Findings from Protocol-Based Subgroup Analyses
Source: PLoS One. 2016 Apr 5;11(4):e0152423. doi: 10.1371/journal.pone.0152423 (PMC4821581; doi:10.1371/journal.pone.0152423)
Supplement: S1 Text — (PDF) [file pone.0152423.s003.pdf]

Supplementary Materials: Power analysis

Method

We used a Monte Carlo approach to explore the statistical power to detect moderation effects given the sample sizes that could hope to be achieved and plausible population parameters for the statistical model judged appropriate for our research questions. The Monte Carlo approach involves specifying a population model, generating random data conforming to this model for a large number of replications and fitting the model of interest to each of these datasets. The proportion of these simulated datasets in which the null hypothesis that a parameter of interest is not equal to zero is correctly rejected serves as an estimate of statistical power.

Population model

As our population model, we took:

$$\eta_{ij} = \gamma_{01}Treat_j + u_{0j} + \beta_1 BaseExcl_{ij} + \beta_2 Moderator_{ij} + \beta_3 Moderator_{ij} \times Treat_{ij} \tag{1}$$

$$\tilde{y}_{ij} = \eta_{ij} + \varepsilon_{ij} \tag{2}$$

where $BaseExcl_{ij}$ is exclusion at baseline for individual *i* in school *j*, $Moderator_{ij}$ is the candidate moderator variable for individual *i* in school *j* and $Moderator_{ij} \times Treat_{ij}$ is a product term formed of the moderator variable and the treatment

allocation received by individual *i* in school *j*; β_1 is the standardised fixed main effect for exclusion at baseline; β_2 is the standardised fixed main effect of the candidate moderator; β_3 is the fixed moderation effect of the candidate moderator. The decision to treat β_3 as an individual level effect is discussed in detail in the 'Method' section of the manuscript. In addition, $Treat_j$ is the treatment variable, here a level-2 predictor, γ_{01} is the effect of treatment on school intercept and u_{0j} is a school-level residual where $u_{0j} \sim N(0, {\sigma_u}^2)$. The variable $\tilde{y}_{ij}$ is a continuous variate underlying the observed outcome variable $Excl_{ij}$. When $\tilde{y}_{ij}$ crosses a threshold=0, $Excl_{ij}$=1 and otherwise $Excl_{ij}$=0. The individual-level residual is ε_{ij} ~logistic(0, $\frac{\pi^2}{3}$) i.e. distributed according to the standard logistic distribution. The key parameter of interest is β_3, capturing the moderation of treatment by the candidate moderators.

Population parameters

We used a fully crossed design to evaluate expected power across a range of plausible population parameter and level-1 and level-2 sample size conditions. A maximum number of 40 level-2 units with 24 level-1 units per level-2 unit was imposed based on financial and logistical limits. We evaluated power at this maximum level-1 and level-2 sample as well as two recruitment and retention fell short of this: for 34 level-2 units with 20 level-1 units per level-2 unit and for 30 level-2 units with 18 level-1 units per level-2 unit. We varied the population moderation parameterβ_3, estimating power for $\beta_3 = .15$, .20 and .25. We judged the minimum clinically meaningful size of β_3 to be .15, therefore, we evaluated power for effects of this magnitude and larger only. We also varied the population intraclass correlation coefficient (ICC), estimating power for ICC=.05 and ICC=.15. We treated all other population parameters as fixed across conditions setting β_1=0.5, β_2=0.2, and

$\gamma_{01} = .1$. $\sigma_u{}^2$ was fixed according to the desired ICC. These conditions are summarised in Table 1.

Model fitting

We fit the true model to the data (i.e. the population model presented in equations 1 and 2 over 1000 replications to give a Monte Carlo sample size of N=1000. Power was estimated as the proportion of replications for which the null hypothesis that $B_3 = 0$ is correctly rejected for alpha=.05.

Results

Results are provided in Table 1. Power was close to or greater than .80 when the magnitude of β_3 was around .20-.25. This suggests that the study had good power to detect a moderation effect of this magnitude and above, however, a lack of significant moderation would not be strong evidence against there being moderation effects smaller than this.

Table A: Monte Carlo study results

β_3 population value	ICC	N level-2 units	Level-1 per level-2 unit	Power
.15	.15	40	24	.55
.20	.15	40	24	.78
.25	.15	40	24	.92
.15	.15	34	20	.42
.20	.15	34	20	.64
.25	.15	34	20	.82
.15	.15	30	18	.32
.20	.15	30	18	.52
.25	.15	30	18	.74
.15	.05	40	24	.56
.20	.05	40	24	.83
.25	.05	40	24	.94
.15	.05	34	20	.46
.20	.05	34	20	.70
.25	.05	34	20	.85
.15	.05	30	18	.36
.20	.05	30	18	.56
.25	.05	30	18	.77
